# Consecutive SSCs increase the SSC effect in skinned rat muscle fibres

**DOI:** 10.1007/s00424-025-03088-2

**Published:** 2025-05-08

**Authors:** Tobias Elst, Sven Weidner, André Tomalka, Daniel Hahn, Florian Kurt Paternoster, Wolfgang Seiberl, Tobias Siebert

**Affiliations:** 1 Motion and Exercise Science, University of Stuttgart, Stuttgart, Germany; 2 Human Movement Science, Faculty of Sports Science, Ruhr University Bochum, Bochum, Germany; 3 School of Human Movement and Nutrition Sciences, University of Queensland, Brisbane, QLD Australia; 4 Biomechanics in Sports, Department of Sport and Health Sciences, Technical University of Munich, Munich, Germany; 5 Human Movement Science, University of the Bundeswehr Munich, Neubiberg, Germany; 6 Stuttgart Center for Simulation Science, University of Stuttgart, Stuttgart, Germany

**Keywords:** Stretch–shortening cycle, History dependence, Isometric contraction phase, Muscle stretching, Performance enhancement, Mechanosensing

## Abstract

Muscle function is essential for generating force and movement, with stretch–shortening cycles (SSCs) playing a fundamental role in the economy of everyday locomotion. Compared with pure shortening contractions, the SSC effect is characterised by increased force, work, and power output during the SSC shortening phase. Few studies have investigated whether SSC effects transfer across consecutive SSCs. Therefore, we investigated SSC effects over three consecutive SSCs in skinned rat muscle fibres by analysing the isometric force immediately before stretch onset (*F*_onset_), the peak force at the end of stretching (*F*_peak_), and the minimum force at the end of shortening (*F*_min_), along with mechanical (*Work*_SSC_) and shortening work (*Work*_SHO_) at different activation levels (20%, 60%, and 100%). Each SSC was followed by an isometric hold phase, allowing force to return to a steady state. Results indicated an increase in both *F*_peak_ (20.3%) and *Work*_SSC_ (60.9%) from SSC1 to SSC3 across all activation levels tested. At 20% and 60% activation, *F*_onset_, *F*_min_*,* and *Work*_SHO_ increased (range: 4.5–28.5%) from SSC1 to SSC3. However, at 100% activation, *F*_onset_ and *Work*_SHO_ remained unchanged, while *F*_min_ decreased (− 8.5%) from SSC1 to SSC3. These results suggest that the increase in SSC effects at submaximal activation may be primarily due to increased cross-bridge forces. The absence of increases in *F*_onset_, *F*_min_, and *Work*_SHO_ at 100% activation suggests that increases in *F*_peak_ and *Work*_SSC_ may not be attributed to increased cross-bridge force but could instead be caused by additional effects, possibly involving modulation of non-cross-bridge structures, likely titin, and their stiffness.

**Supplementary Information:**

The online version contains supplementary material available at 10.1007/s00424-025-03088-2.

## Introduction

Muscle function is a complex interplay of biochemical and biomechanical processes that enable force generation and movement. Among the countless mechanisms that modulate muscle force, the stretch–shortening cycle (SSC) stands out as a fundamental contraction type [17, 56, 88, 89] with profound implications for athletic performance, everyday locomotion, rehabilitation, and injury prevention [57, 100, 105]. The SSC, characterised by an active muscle stretch followed by an immediate shortening contraction [17], underlies a variety of dynamic movements in sports and everyday activities such as jumping, running, and walking [56, 75].

Previous studies on SSCs reported a performance enhancement known as the SSC effect during the SSC shortening phase compared with a pure shortening contraction. This SSC effect leads to significantly increased force, work, and power output [9, 17, 34, 97, 99] following active stretches and is often associated with improved energy efficiency and reduced metabolic cost compared with pure shortening contractions [17, 47].

The underlying mechanisms responsible for the SSC effect remain a subject of debate [32, 87]. Explanations for the SSC effect focused on altered neuromuscular activation [8], stretch-reflex responses [23], or elastic recoil from serial elastic tissues [48, 53]. These theories, however, have been challenged by findings that demonstrate the SSC effect at the level of isolated muscle fibres, where none of the mentioned mechanisms can account for the observed effects [17, 28, 44, 97].

As a consequence, approaches that model muscle force at the cellular level beyond the known force–length [33] and force–velocity dependencies [49] need to be explored and discussed. This involves the history-dependency of muscle force, including residual force enhancement following active muscle lengthening and residual force depression following active muscle shortening [1]. There is experimental [24, 97] and modelling [77, 83, 86] evidence that suggests that residual force enhancement is induced by a calcium-dependent parallel elastic element within the sarcomere (titin), modifying its stiffness upon activation during lengthening. The spring-like protein titin is implicated, but it has proven difficult to study its function directly. Residual force depression presumably results from inhibited cross-bridge attachments [69] or a contribution of the activation-dependent titin-actin interaction during muscle shortening [83, 86, 93]. Both history-dependent effects (residual force enhancement and residual force depression) occur under maximal and submaximal conditions, with lengthening-induced residual force enhancement and shortening-induced residual force depression being differentially affected by submaximal activation, suggesting distinct underlying mechanisms [71]. Skeletal muscle titin is known to become stiffer upon muscle activation in the presence of Ca^2+^ (calcium-responsive) [58]. More important, however, is the property of skeletal muscle titin to significantly reduce its persistence length upon activation (by potential titin-actin interactions) and thus to produce increased forces during subsequent stretching [24, 52, 54, 58, 93]. Especially in the last decade, a series of SSC studies focused on the history-dependent properties of stretch-induced force enhancement, emphasising the roles of cross-bridge dynamics, non-cross-bridge structures, and residual force enhancement in SSC performance, providing strong support for their relevance in SSCs [29, 30, 37, 97]. History-dependent effects influence the transient phases of stretch and shortening (e.g. [18, 79]) as well as the force following these contractions (e.g. [89, 99]). Consequently, these effects likely play a role in SSC transitions, affecting both individual and consecutive SSCs, as the isometric force before and after each SSC in a series of consecutive SSCs is expected to differ.

In addition to these findings, recent investigations have proposed that cross-bridge force is not solely controlled by calcium activation of the actin-containing thin filament but rather by a dual process that also involves activation of the myosin-containing thick filament as well [10, 50, 64, 68]. X-ray diffraction studies of actively contracting skeletal muscles have revealed that myosin filaments can exist in multiple states [10, 64, 74], defined by the conformation of their myosin heads. Myosin heads can adopt two structural states: the ‘ON’ state or the ‘OFF’ state. In the ON state, myosin heads are positioned away from the thick filament backbone and thus able to bind to thin filaments during contraction. Conversely, in the OFF state, myosin heads are docked near the thick filament backbone in quasi-helical arrangements, which prevent them from interacting with actin [20, 50, 70]. Alongside the ‘ON’ and ‘OFF’ states, myosin heads transition along a spectrum between these two states, with their propensity to form cross-bridges upon activation depending on their position within this range. It is assumed that various physiological and biomechanical factors regulate these states and transitions [41, 74]. During the SSC, changes in the underlying ON/OFF states of myosin heads could occur [41, 74], leading to variations in cross-bridge formation, which may influence the SSC effect.

While previous research focused on single SSCs to characterise the SSC effect and its underpinning mechanisms during one cycle, real-world activities often involve multiple consecutive SSCs. For instance, activities like strength training or sports such as alpine skiing require continuous muscle activation over multiple consecutive SSCs [7, 19]. However, there is limited understanding of how the SSC effect develops over a sequence of cycles and whether history-dependent effects accumulate over time.

**Fig. 1:**
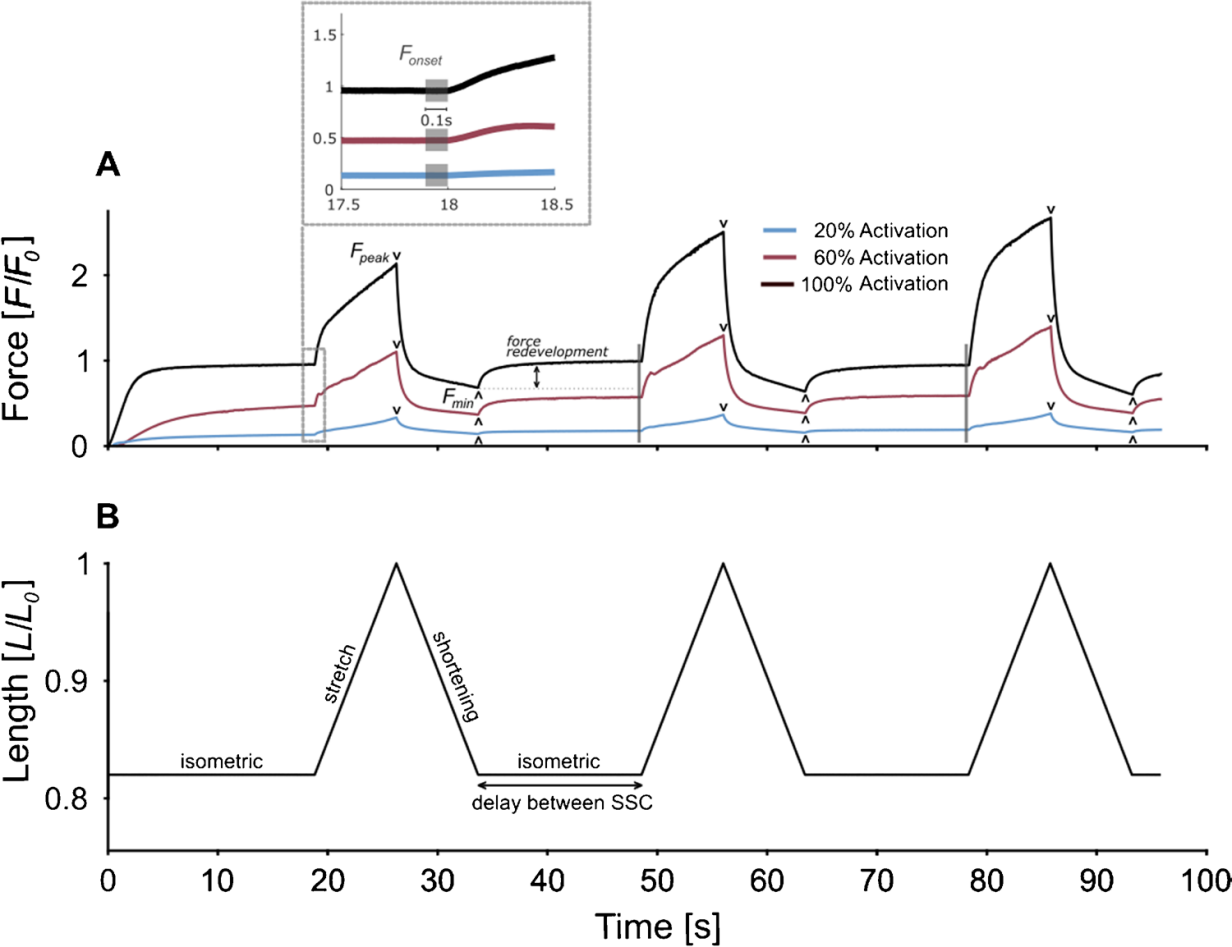
Exemplary dataset of force–time and length–time traces for an activated muscle fibre. **A** Normalised force (*F*/*F*_0_) over time (s) of a single-skinned extensor digitorum longus (EDL) muscle fibre at three different activation levels. Activation levels are indicated by different colours: 100% (black), 60% (red), and 20% (blue) of maximum activation. Three stretch–shortening cycles (SSCs) were performed during one continuous activation period. The fibres were activated at second 0 and remained active for ~ 96 s until shortly after the third SSC. Each SSC consisted of a stretch and a shortening phase with a stretch amplitude of 0.18 *L*_0_ and a stretch velocity of 1% *v*_max_. The isometric contraction phase between two cycles is of the same duration as one SSC. Steady-state force (*F*_onset_, grey shaded 0.1-s interval, see zoom in) at the end of an isometric contraction phase, peak force (*F*_peak_, black ‘v’) at the end of the SSC lengthening phase, and minimum force (*F*_min_, black ‘ʌ’) at the end of the SSC shortening phase were marked for all SSCs in (**A**) and additionally labelled for the first SSC. Force redevelopment describes the force recovery in the isometric contraction phase that follows the shortening phase and is indicated after the first SSC for 100% activation. **B** Normalised length (*L*/*L*_0_) time traces of the performed SSCs. The delay describes the duration of isometric contraction between two individual SSCs

Studies on isolated muscles and muscle fibres with sustained activation across multiple SSCs indicate that the delay between individual SSCs (c.f. Figure 1B), during which the fibres contract isometrically, affects the force response [3, 13, 14, 44, 61]. A longer delay between SSCs with continuous activation allows the muscle to redevelop force to an isometric steady state after the active shortening phase of each SSC. This phenomenon is known as force redevelopment (see Fig. 1A). To better investigate how SSC effects change across consecutive SSCs, our study focused on using a delay (~ 15 s), which ensures that each SSC begins from an isometric steady state and thus under comparable conditions [14]. In particular, this study analysed forces immediately before stretch onset of an SSC (*F*_onset_), at the end of the SSC stretching phase (*F*_peak_), and at the end of the SSC shortening phase (*F*_min_) (refer to Fig. 1A), as well as work output including both the absolute work of the entire SSC (*Work*_SSC_) and the work during the shortening phase of the SSC (*Work*_SHO_) (refer to Fig. 3). Changes in force and work parameters were examined across three consecutive SSCs at three different activation levels (20%, 60%, and 100% of maximum activation). Given that Campbell and Moss [14] applied longer fixed-end contractions between two consecutive SSCs, leading to increased *F*_onset_ and *F*_peak_ values, and Andersen et al. [5] reported a similar increase in *F*_peak_ under comparable conditions, we expected to observe a similar behaviour in our study. Specifically, we expected a cumulative effect for fixed-end contractions and SSCs of equal duration, with both force and work progressively increasing with each SSC across all activation levels tested.

## Methods

### Animal and tissue preparation

Extensor digitorum longus (EDL) muscles were harvested from three male Wistar rats (8–10 months old, 300–350 g) maintained under a 12-h light/dark cycle at 22 °C. The animals were euthanised using a CO_2_ overdose. Our procedures adhered to the standards of ARRIVE guidelines and were approved under the German animal protection law (Tierschutzgesetz, §4 (3); Permit No. T 201/21 ST).

EDL muscles, extracted from the three left hind limbs, were processed following the protocol described by Tomalka et al. [96, 98]. Briefly, muscle fibres (*n* = 24) were dissected, permeabilised in a skinning solution (see below), and then stored in a 50/50 glycerol-skinning solution mixture at − 20 °C for 6–8 weeks. On experimentation days, single fibres were dissected under a dissecting microscope using fine forceps. Then, muscle fibres were cut to a length of 1.02 ± 0.15 mm and clamped on both sides with aluminium foil T-shaped clips. To ensure complete removal of internal fibre membranes, we treated the fibre preparations with a Triton X-100 enriched skinning solution (1% vol/vol Triton X-100 in relaxation solution) for approximately 2–3 min at 4 °C.

### Solutions

The relaxing solution contained (in mM) 100 TES, 7.7 MgCl_2_, 5.44 Na_2_ATP, 25 EGTA, 19.11 Na_2_CP, and 10 glutathione (GLH) (pCa 9.0). The preactivating solution contained (in mM) 100 TES, 6.93 MgCl_2_, 5.45 Na_2_ATP, 0.1 EGTA, 19.49 Na_2_CP, 10 GLH, and 24.9 1,6-diaminohexane-N,N,N′,N′-tetraacetic acid (HDTA). The activating solution contained 100 TES, 6.76 MgCl_2_, 5.46 Na_2_ATP, 19.49 Na_2_CP, 10 GLH, and 25 CaEGTA (pCa 4.5), and the skinning solution contained 170 potassium propionate, 2.5 MgCl_2_, 2.5 Na_2_ATP, 5 EGTA, 10 imidazole, and 0.2 PMSF. The storage solution is the same as the skinning solution, except for the presence of 10 mM GLH and 50% glycerol (vol/vol). Cysteine and cysteine/serine protease inhibitors (trans-epoxysuccinyl-L leucylamido-(4-guanidino) butane, E-64, 10 mM; leupeptin, 20 µg·mL^−1^) were added to all solutions to preserve lattice proteins and thus sarcomere homogeneity [65, 96], thereby minimising changes in regional variations in sarcomere length [94]. pH (adjusted with potassium hydroxide) was 7.1 at 12 °C. Subsequently, all solutions, except for the skinning and storage solutions, were supplemented with 450 U·mL^−1^ of creatine kinase (CK) on the experimental day. The CK used in these experiments was sourced from Roche (Mannheim, Germany). As for the other chemicals utilised in our study, they were procured from Sigma (St. Louis, MO, USA). The solutions used throughout the experiments were identical to those described in Linari et al. [65].

### Experimental setup

Initially, muscle fibres were transitioned from the skinning solution to the experimental chamber of the fibre-test apparatus (Model 1400 A, Aurora Scientific, Canada). Subsequently, the fibre-clip assembly was secured to both a force transducer (Model 403 A, Aurora Scientific, Ontario, Canada) and a length controller (Model 322 C-I, Aurora Scientific, Ontario, Canada). This apparatus was then positioned on the x–y moving stage of an inverted microscope (Eclipse Ti-S, Nikon, Japan) for detailed observation and manipulation. To ensure stability and optimal mechanical performance during experiments, the T-clips were securely adhered to the hooks of the force transducer and length controller using fingernail polish, diluted with acetone.

Measurements of sarcomere length were carried out in the central segment of each fibre. The optimal sarcomere length, at which the fibre produces maximum force (*F*_0_), was determined to be 2.5 ± 0.05 µm (mean ± standard deviation) in passive state [92, 96]. This length was referred to as optimal length (*L*_opt_). For precise morphological analysis, both the width (*w*) and height (*h*) of the fibres were gauged at intervals of 0.1 mm. The fibres’ cross-sectional area, assuming an elliptical shape, was calculated as *π* × *h* × *w*/4 and was found to be 0.0043 ± 0.0017 mm^2^.

### Experimental protocol

All skinned fibre experiments were conducted at a constant solution temperature of 12 ± 0.1 °C, as this temperature provided stability for the fibres during active lengthening and prolonged activations [81, 82]. The activation of fibres involved three steps in the presence of ATP.

(1) During preactivation, fibres were immersed in a preactivating solution (pCa 9.0) for 60 s for equilibration. (2) The fibres were then transferred to an activation solution, leading to a rapid increase in force until a stable steady-state plateau was reached. After an initial isometric contraction of 18 s, three SSCs were performed (Fig. 1). Activation levels during step (2) were 20% (pCa 6.34), 60% (pCa 6.08), and 100% (pCa 4.45) of activation [6]. (3) Lastly, the fibres were immersed in a relaxing solution (pCa 9.0) for at least 420 s.

The experimental protocol consisted of performing two fixed-end contractions both before and after three SSC sets, adding up to a total of 7 activations per fibre. First, a fixed-end reference contraction (100% activation) was conducted at 1.0 *L*_opt_, followed by a second fixed-end contraction (100% activation) at 0.82 *L*_opt_. Afterwards, three sets of SSCs were performed, each set under a different activation condition (20%, 60%, or 100% activation). The order of the SSC sets at different activation levels (20%, 60%, or 100% activation) was randomised to prevent systematic effects of fatigue on the force parameters. Each SSC set involved three consecutive SSCs that were separated by an isometric hold phase (to reach a steady-state force before each SSC onset) (Fig. 1). SSCs were conducted from 0.82 to 1.0 back to 0.82 *L*_opt_ (see Fig. 1), which is within the typical rat EDL muscle working range [12]. The SSCs were conducted at 1% of the maximum shortening velocity (*v*_max_) in accordance with Weidner et al. [103, 104]. The activation process for a SSC set began after the fibre length was passively set to 0.82 *L*_opt_. The fibres were continuously activated throughout the entire three SSCs within a set, including the isometric hold phases. An isometric hold phase between SSCs was of the same duration as one SSC itself (which is approximately 14.9 s) (Fig. 1).

Once the three sets of SSC contractions were completed, two fixed-end contractions were repeated in reversed order, first at 0.82 *L*_opt_, then at 1.0 *L*_opt_. The steady-state forces measured during the reference contractions at 1.0 *L*_opt_ were averaged and used to normalise the force data for that specific fibre. During activation of the fixed-end reference contractions, sarcomere length decreased from 2.49 ± 0.04 µm in the passive state to 2.21 ± 0.17 µm in the active state. In our experiments, isometric force in successive activations decreased at an average rate of 2.79 ± 0.91% per activation, up to a maximum of 7 activations per fibre. This rate of force loss agrees with other studies under similar conditions [21, 96], indicating consistent preparation routines and fibre functionality.

### Data processing and statistics

Data acquisition involved the recording of force, the position of the length controller, and solution temperature at a sampling rate of 1000 Hz using an A/D interface (604 A, Aurora Scientific, Canada) and real-time software (600 A, Aurora Scientific). Subsequent data analysis was performed using a custom-written MATLAB (v2023b; MathWorks, Natick, MA, USA) script and Excel (v1808; Microsoft Corporation, Redmond, WA, USA). Maximum muscle force (*F*/*F*_0_) and optimal fibre length (*L*/*L*_0_) were normalised to each fibre. The shortening velocity was normalised to the muscle-specific maximum shortening velocity (*v*/*v*_max_). The *v*_max_ of skinned EDL muscle fibres was determined to be 2.42 ± 0.08 *L*_opt_ s^−1^ in a separate set of fibres (*n* = 5 fibres) at 100% activation (pCa 4.5), following the methodology of Tomalka et al. [98] and supported by literature data [22, 96]. Forces immediately before stretch onset (*F*_onset_) at the end of the isometric steady state (Fig. 1) were calculated as the mean force of a 0.1-s interval. For each SSC, peak forces at the end of stretching (*F*_peak_) and minimum forces at the end of shortening (*F*_min_) were determined as the highest and lowest values, respectively, within an SSC interval (Fig. 1), using the MATLAB functions ‘max’ and ‘min’. *F*_onset_*, F*_peak_*,* and *F*_min_ were normalised to *F*_0_. Mechanical work during shortening (*Work*_SHO_; c.f. Figure 3B) was defined as the ‘positive work’ performed during the active shortening phase of the SSC. Mechanical work during the SSC (*Work*_SSC_; c.f. Figure 3B) was determined by subtracting *Work*_SHO_ from the absolute amount of work during lengthening. *Work*_SSC_ corresponds to the area within the work loop. Work is numerically accomplished by integrating the force over the length of the fibre using the trapezoidal method, implemented through the MATLAB function ‘trapz’. Unless stated otherwise, work is expressed in normalised values (∫*F*/*F*_0_ × *∆L*/*L*_0_). To examine changes in a parameter (e.g. *F*_peak_*)* between SSCs at a given calcium concentration, we calculated the differences between mean values, expressed as $({F}_{\text{peak}\_\text{SSC}3}-{F}_{\text{peak}\_\text{SSC}1})$ [*F*_0_], and the relative differences as $\left(\frac{{F}_{\text{peak}\_\text{SSC}3}-{F}_{\text{peak}\_\text{SSC}1}}{{F}_{\text{peak}\_\text{SSC}1}}\right)$ [% *F*_0_]. Similar calculations were performed for the remaining parameters. Data sets within an interquartile range of 3 were used for evaluation. Statistics for 60% and 100% activation were built on 24 individual muscle fibres. For 20% activation, only 22 of the 24 fibres were used for statistical analysis, since two fibres were immersed in the incorrect activating solution. Mauchly tests were conducted to ensure sphericity. Related QQ plots are available in the Supplementary Information (Fig. S3). Repeated measures (RM) ANOVAs were calculated to test whether *F*_onset_, *F*_peak_, *F*_min_*, Work*_SSC_, or *Work*_SHO_ differed between the three SSCs within each activation level. Each RM ANOVA was corrected with a Greenhouse–Geisser adjustment if sphericity was not given. When ANOVAs revealed significant differences, post hoc analyses were performed with pairwise comparisons between groups. Exact *p*-values were reported for interpretation of the data. Statistical analyses were performed using SPSS version 29 (IBM Corp., Armonk, NY, USA), with the significance level set at *p* = 0.05. Figures and tables were created using MATLAB (v2023b; MathWorks, Natick, MA, USA), Excel (v1808; Microsoft Corporation, Redmond, WA, USA), and Inkscape (v1.3.2; Inkscape Project, Brooklyn, NY, USA).

## Results

Tested muscle fibres (*n* = 24) generated a maximum isometric stress of 78.2 ± 20.8 kN·m^−2^ at optimal length and full activation. Active muscle stretches led to instantaneously increasing forces, resulting in a distinct force peak at the end of the stretch (Fig. 1, *F*_peak_). During the SSC shortening phase, the fibre was returned to its starting length, and force fell to a local minimum (Fig. 1, *F*_min_). As soon as shortening ceased, force redeveloped as expected during the isometric phase in between the cycles, leading to a steady state (Fig. 1, *F*_onset_) upon the initiation of the subsequent cycle. On average, *F*_onset_ and *F*_peak_ were 15.7% *F*_0_ and 28.1% *F*_0_ at 20% activation, 57.5% *F*_0_ and 107.5% *F*_0_ at 60% activation, and 87.6% *F*_0_ and 189.7% *F*_0_ at 100% activation. Detailed descriptive and statistical analysis of *F*_onset_, *F*_peak_, *F*_min_, *Work*_SSC_, and *Work*_SHO_ are summarised in Tables 1 and 2.

**Table 1:** Descriptive statistics and pairwise comparison of onset force (*F*_onset_), peak force (*F*_peak_), and minimum force (*F*_min_). For *F*_onset_ at 100% activation, ANOVA did not indicate a significant main effect. Consequently, no post hoc tests were performed, and *p*-values are not reported

Activation (%)	Cycle	Descriptive statistics		Comparison of	Pairwise comparison				*p*-values
					Differences of mean values		95% confidence interval of the difference		
		Mean	SD		Total	Relative	Lower	Upper	
**Onset force (*F***_**onset**_**)**		**(*F***_**onset**_**/*F***_**0**_**)**			**(*F***_**0**_**)**	**(%)**			
20	SSC1	0.134	0.069	SSC1 → SSC2	0.029	22.0	0.022	0.037	< 0.001
20	SSC2	0.164	0.076	SSC2 → SSC3	0.009	6.6	0.005	0.013	< 0.001
20	SSC3	0.172	0.079	SSC1 → SSC3	0.038	28.5	0.028	0.049	< 0.001
60	SSC1	0.512	0.131	SSC1 → SSC2	0.089	17.5	0.068	0.111	< 0.001
60	SSC2	0.601	0.142	SSC2 → SSC3	0.015	3.0	0.009	0.022	< 0.001
60	SSC3	0.617	0.139	SSC1 → SSC3	0.105	20.4	0.078	0.131	< 0.001
100	SSC1	0.869	0.083	SSC1 → SSC2	0.025	2.9	–-	–-	–-
100	SSC2	0.894	0.094	SSC2 → SSC3	− 0.027	− 3.1	–-	–-	–-
100	SSC3	0.867	0.102	SSC1 → SSC3	− 0.002	− 0.3	–-	–-	–-
**Peak force (*F***_**peak**_**)**		**(*F***_**peak**_**/*F***_**0**_**)**			**(*F***_**0**_**)**	**(%)**			
20	SSC1	0.261	0.094	SSC1 → SSC2	0.026	10.1	0.017	0.035	< 0.001
20	SSC2	0.288	0.101	SSC2 → SSC3	0.007	2.6	0.003	0.010	< 0.001
20	SSC3	0.294	0.103	SSC1 → SSC3	0.033	12.7	0.021	0.045	< 0.001
60	SSC1	0.922	0.227	SSC1 → SSC2	0.182	19.7	0.148	0.216	< 0.001
60	SSC2	1.104	0.278	SSC2 → SSC3	0.097	10.6	0.073	0.122	< 0.001
60	SSC3	1.201	0.313	SSC1 → SSC3	0.279	30.3	0.223	0.335	< 0.001
100	SSC1	1.721	0.320	SSC1 → SSC2	0.218	12.7	0.164	0.273	< 0.001
100	SSC2	1.939	0.436	SSC2 → SSC3	0.092	5.3	0.064	0.120	< 0.001
100	SSC3	2.031	0.496	SSC1 → SSC3	0.310	18.0	0.228	0.392	< 0.001
**Minimum force (*F***_**min**_**)**		**(*F***_**min**_**/*F***_**0**_**)**			**(*F***_**0**_**)**	**(%)**			
20	SSC1	0.113	0.056	SSC1 → SSC2	0.014	12.3	0.010	0.018	< 0.001
20	SSC2	0.126	0.056	SSC2 → SSC3	0.003	3.0	0.002	0.005	< 0.001
20	SSC3	0.130	0.057	SSC1 → SSC3	0.017	15.4	0.012	0.022	< 0.001
60	SSC1	0.411	0.103	SSC1 → SSC2	0.018	4.3	0.011	0.025	< 0.001
60	SSC2	0.429	0.101	SSC2 → SSC3	0.001	0.3	− 0.003	0.005	0.573
60	SSC3	0.430	0.100	SSC1 → SSC3	0.019	4.5	0.008	0.029	0.001
100	SSC1	0.644	0.081	SSC1 → SSC2	− 0.029	− 4.6	− 0.037	− 0.022	< 0.001
100	SSC2	0.614	0.085	SSC2 → SSC3	− 0.025	− 3.9	− 0.030	− 0.021	< 0.001
100	SSC3	0.589	0.087	SSC1 → SSC3	− 0.055	− 8.5	− 0.067	− 0.043	< 0.001

**Table 2:** Descriptive statistics and pairwise comparison of absolute work of the entire SSC (*Work*_SSC_) and shortening work (*Work*_SHO_) per cycle. For *Work*_SHO_ at 100% activation, ANOVA did not indicate a significant main effect. Consequently, no post hoc tests were performed, and *p*-values are not reported

Activation (%)	Cycle	Descriptive statistics		Comparison of	Pairwise comparison				*p*-values
					Differences of mean values		95% confidence interval of the difference		
		Mean	SD		Total	Relative	Lower	Upper	
***Work***_**SSC**_		**(∫*F*/*F***_**0**_** × ∆*L*/*L***_**opt**_** × 10**^**3**^**)**			**(∫*F*/*F***_**0**_** × ∆*L*/*L***_**opt**_** × 10**^**3**^**)**	**(%)**			
20	SSC1	7.7	5.2	SSC1 → SSC2	2.6	33.4	1.9	3.3	< 0.001
20	SSC2	10.3	5.8	SSC2 → SSC3	0.9	11.7	0.5	1.3	< 0.001
20	SSC3	11.2	6.0	SSC1 → SSC3	3.5	45.2	2.5	4.5	< 0.001
60	SSC1	42.4	18.9	SSC1 → SSC2	23.3	55.0	18.9	27.7	< 0.001
60	SSC2	65.7	25.9	SSC2 → SSC3	13.2	31.2	10.0	16.5	< 0.001
60	SSC3	78.9	31.4	SSC1 → SSC3	36.5	86.2	29.2	43.9	< 0.001
100	SSC1	112.4	30.8	SSC1 → SSC2	41.6	37.0	32.5	50.7	< 0.001
100	SSC2	154.0	49.9	SSC2 → SSC3	16.3	14.5	11.8	20.7	< 0.001
100	SSC3	170.3	58.8	SSC1 → SSC3	57.9	51.5	44.4	71.3	< 0.001
***Work***_**SHO**_		**(∫*F*/*F***_**0**_** × ∆*L*/*L***_**opt**_** × 10**^**3**^**)**			**(∫*F*/*F***_**0**_** × ∆*L*/*L***_**opt**_** × 10**^**3**^**)**	**(%)**			
20	SSC1	27.3	10.8	SSC1 → SSC2	2.8	10.3	2.0	3.6	< 0.001
20	SSC2	30.1	11.2	SSC2 → SSC3	0.8	3.0	0.5	1.2	< 0.001
20	SSC3	30.9	11.5	SSC1 → SSC3	3.6	13.3	2.5	4.7	< 0.001
60	SSC1	91.3	18.3	SSC1 → SSC2	7.5	8.2	6.1	8.9	< 0.001
60	SSC2	98.8	17.8	SSC2 → SSC3	2.5	2.7	1.7	3.3	< 0.001
60	SSC3	101.3	17.4	SSC1 → SSC3	10.0	10.9	7.8	12.1	< 0.001
100	SSC1	146.1	13.0	SSC1 → SSC2	2.0	1.4	–-	–-	–-
100	SSC2	148.1	14.7	SSC2 → SSC3	− 0.9	− 0.6	–-	–-	–-
100	SSC3	147.2	15.7	SSC1 → SSC3	1.1	0.7	–-	–-	–-

### Changes in *F*_onset_

**Fig. 2:**
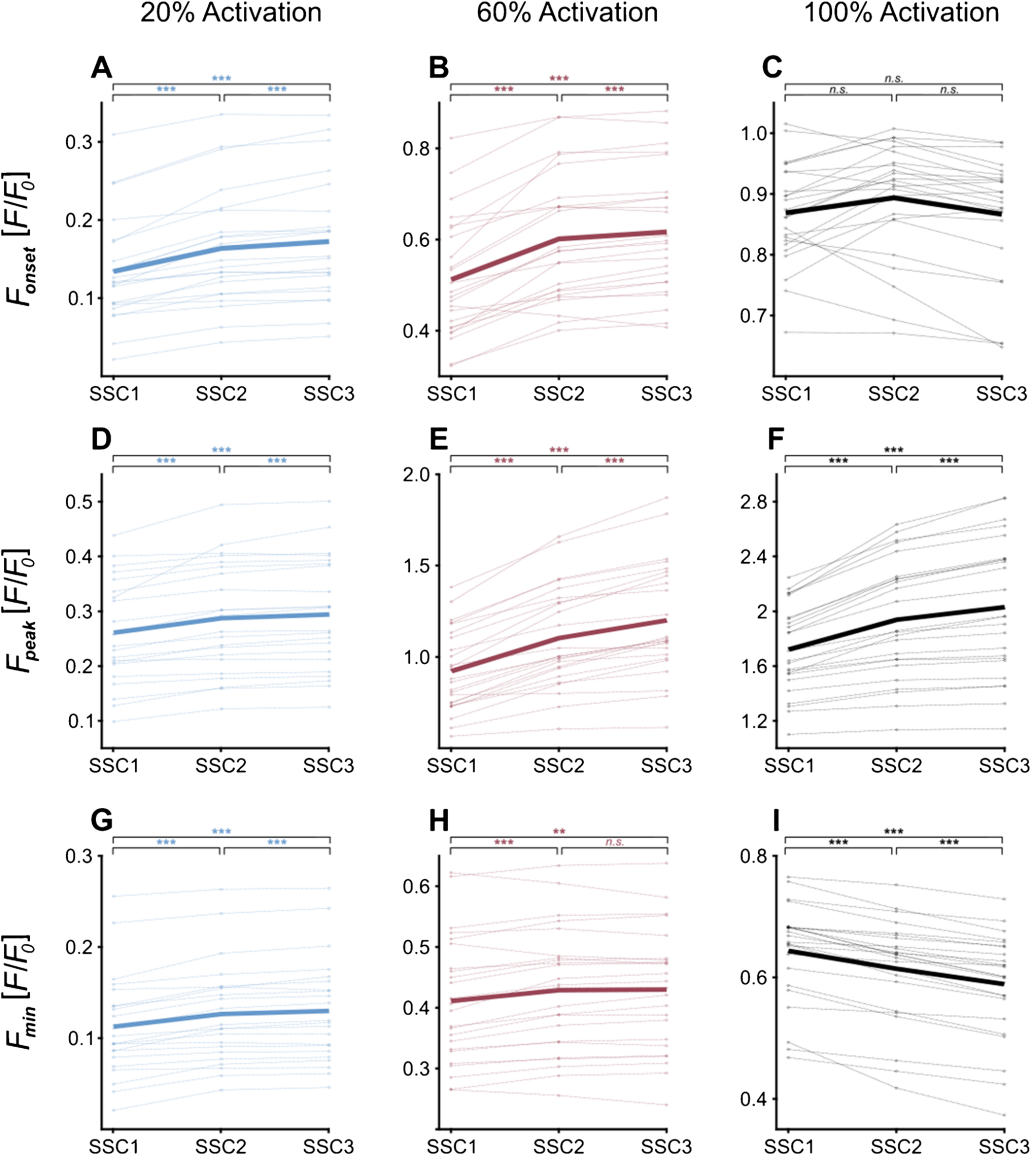
Comparative analysis of force immediately before stretch onset (*F*_onset_), peak force at the end of stretching (*F*_peak_), and minimum force at the end of shortening (*F*_min_) across three consecutive stretch–shortening cycles (SSCs) at varying activation levels. The *y*-axis represents normalised force (*F*/*F*_0_), while the *x*-axis indicates the numbers of the three consecutive cycles (SSC1, SSC2, and SSC3). Each subplot contains 24 individual data (except for 20% activation, where only 22 fibres were analysed). The bold lines represent the mean values. Significance levels are indicated using asterisks: ***, denoting *p* < 0.001; **, indicating *p* < 0.01; and n.s., indicating no significant differences. **A** The *F*_onset_ dataset for 20% activation, **B** the activation level of 60%, and **C** the activation level of 100%. *F*_peak_ datasets **D** for 20% activation, **E** for 60% activation, and **F** for 100% activation. Finally, the *F*_min_ dataset for **G** 20% activation, **H** for 60% activation, and **I** for 100% activation. Note the different scaling of each *y*-axis. *F*_onset_ increases from SSC1 to SSC3 at 20% and 60% activation, while no differences are present at 100% activation. *F*_peak_ increases from cycle to cycle within each activation level tested. *F*_min_ increases from SSC1 to SSC3 at 20% and 60% activation while it decreases from SSC1 to SSC3 at 100% activation

Repeated measures ANOVA showed differences in *F*_onset_ between SSCs for 20% activation (*n* = 22) [*F*(1.09, 22.80) = 60.57, *p* < 0.001]. Post hoc analysis revealed that *F*_onset_ increased from SSC1 to SSC2 (+ 22.0%, *p* < 0.001), from SSC2 to SSC3 (+ 6.6%, *p* < 0.001), and from SSC1 to SSC3 (+ 28.5%, *p* < 0.001) (Fig. 2A, Table 1). Similar differences in *F*_onset_ were attained for 60% activation (*n* = 24) [*F*(1.05, 24.12) = 67.78, *p* < 0.001]. Pairwise comparisons indicated that *F*_onset_ increased by 17.5% (*p* < 0.001) from SSC1 to SSC2, by 3.0% (*p* < 0.001) from SSC2 to SSC3, and by 20.4% (*p* < 0.001) from SSC1 to SSC3 (Fig. 2B, Table 1). In contrast, *F*_onset_ remained unchanged over the three SSCs for 100% activation, as indicated by the RM ANOVA (*n* = 24) [*F*(1.06, 24.30) = 3.52, *p* = 0.071] (Fig. 2C, Table 1).

### Changes in *F*_peak_

The results of the RM ANOVA showed differences in *F*_peak_ between consecutive SSCs for 20% activation (*n* = 22) [*F*(1.04, 21.88) = 34.69, *p* < 0.001]. Subsequent post hoc analysis revealed that *F*_peak_ increased from SSC1 to SSC2 (+ 10.1%, *p* < 0.001), from SSC2 to SSC3 (+ 2.6%, *p* < 0.01), and from SSC1 to SSC3 (+ 12.7%, *p* < 0.001) (Fig. 2D, Table 1). Similar differences in *F*_peak_ were attained for 60% activation (*n* = 24) [*F*(1.05, 24.10) = 104.96, *p* < 0.001]. Pairwise comparisons indicated that peak force increased by 19.7% (*p* < 0.001) from SSC1 to SSC2, by 10.6% (*p* < 0.001) from SSC2 to SSC3, and by 30.3% (*p* < 0.001) from SSC1 to SSC3 (Fig. 2E, Table 1). Likewise, at 100% activation (*n* = 24) [*F*(1.01, 23.14) = 61.82, *p* < 0.001] analysis showed that *F*_peak_ increased from SSC1 to SSC2 by 12.7% (*p* < 0.001), from SSC2 to SSC3 by 5.3% (*p* < 0.001), and from SSC1 to SSC3 by 18.0% (*p* < 0.001) (Fig. 2F, Table 1). Relative changes in *F*_peak_ depend on activation (*p* < 0.05) (see Fig. S1).

In general, across all activation levels tested, increases in *F*_peak_ are larger (14%) from SSC1 to SSC2 compared to the smaller increase (6%) from SSC2 to SSC3 (Fig. 2 and Table 1).

### Changes in *F*_min_

At 20% activation, RM ANOVA revealed statistical differences (*n* = 22) [*F*(1.06, 22.16) = 52.30, *p* < 0.001], with an increase of 15.4% (*p* < 0.001) in *F*_min_ from SSC1 to SSC3 (Fig. 2G, Table 1). At 60% activation (*n* = 24) [*F*(1.07, 24.69) = 16.64, *p* < 0.001], a 4.5% increase in *F*_min_ (*p* < 0.01) was observed from SSC1 to SSC3 (Fig. 2H, Table 1). Conversely, at 100% activation, multiple comparisons (*n* = 24) [*F*(1.03, 23.70) = 89.50, *p* < 0.001] revealed an 8.5% decrease in *F*_min_ from SSC1 to SSC3 (Fig. 2I, Table 1).

### Changes in work per cycle

**Fig. 3:**
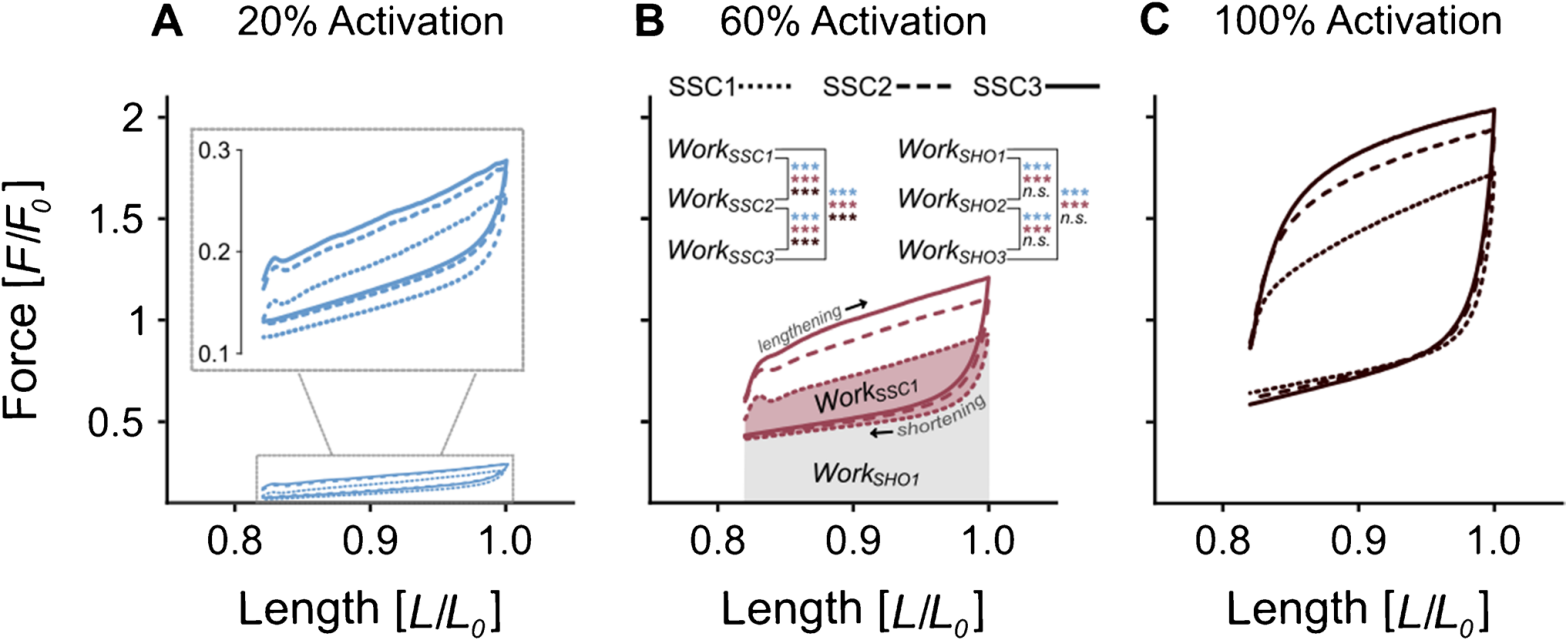
Work loops of stretch–shortening cycles (SSCs) at varying activation levels. The *y*-axis represents normalised force (*F*/*F*_0_), while the *x*-axis indicates the normalised fibre length (*L*/*L*_0_). Each subplot contains the force–length plots of three SSCs performed during one continuous activation. The plots depict the mean values from 24 fibres (except for the 20% activation, which includes only 22 fibres). Significance levels are indicated using asterisks: ***, denoting *p* < 0.001; and n.s., indicating no significant differences. **A** The force–length traces at the lowest activation level of 20%, including a magnified view for detailed analysis. **B** The activation level of 60% alongside the direction of reading the force–length traces (lengthening phase and shortening phase). The area on which the absolute amount of work for the entire SSC (*Work*_SSC_) and the work during the shortening phase of the SSC (*Work*_SHO_) is calculated, is marked exemplarily for the first SSC. **C** The activation level of 100%. Note the equally scaled *y*-axis across all plots. Overall work increases with higher activation levels (**A**–**C**) due to calcium-dependent changes in force production. Moreover, work per loop increases from cycle to cycle at every activation level, showing accumulative effects during one continuous activation period

For the 20% activation condition, *Work*_SSC_ increased [*F*(1.10, 23.05) = 51.31, *p* < 0.001] with each successive SSC. In particular, a 33% increase from SSC1 to SSC2 (dotted vs. dashed line in Fig. 3A, p < 0.001), a 12% increase from SSC2 to SSC3 (dashed vs. solid line in Fig. 3A, p < 0.001), and a 45% increase from SSC1 to SSC3 (dotted vs. solid line in Fig. 3A, p < 0.001) were observed. Similarly, *Work*_SSC_ increased with cycle number for 60% activation [*F*(1.05, 24.16) = 105.27, *p* < 0.001]. Consecutive cycling resulted in a 55% increase in *Work*_SSC_ from SSC1 to SSC2 (dotted vs. dashed line in Fig. 3B, p < 0.001), a 31% increase from SSC2 to SSC3 (dashed vs. solid line in Fig. 3B, p < 0.001), and an 86% increase from SSC1 to SSC3 (dotted vs. solid line in Fig. 3B, p < 0.001). Finally, for 100% activation, *Work*_SSC_ increased [*F*(1.01, 23.25) = 81.02, *p* < 0.001], with a 37% increase from SSC1 to SSC2 (dotted vs. dashed line in Fig. 3C, p < 0.001), a 15% increase from SSC2 to SSC3 (dashed vs. solid line in Fig. 3C, p < 0.001), and a 52% increase from SSC1 to SSC3 (dotted vs. solid line in Fig. 3C, p < 0.001).

*Work*_SHO_ increased by 13.3% (*p* < 0.001) from SSC1 to SSC3 at 20% activation [*F*(1.05, 22.02) = 49.21, *p* < 0.001] and by 10.9% (*p* < 0.001) at 60% activation [*F*(1.05, 24.04) = 97.13, *p* < 0.001]. In contrast, *Work*_SHO_ remained unchanged across the three SSCs at 100% activation [*F*(1.03, 23.58) = 3.49, *p* = 0.073] (Table 2).

## Discussion

In general, this study demonstrated that consecutive SSCs with constant activation and an isometric hold phase between SSCs led to increases in force and work parameters, thereby enhancing SSC performance. However, the magnitude and direction of changes in individual force and work parameters (*F*_onset_, *F*_peak_, *F*_min_, *Work*_SSC_, *and Work*_SHO_) depend on the specific activation condition (Tables 1 and 2). Of particular interest are the changes in *F*_onset_, as they primarily indicate changes in the number of bound cross-bridge in the isometric steady-state phase after the completed SSC. At maximum activation (100%), there was no increase in *F*_onset_ and *Work*_SHO_*,* and a decrease in *F*_min_ was observed from SSC1 to SSC3. Thus, the relative increase of 18% in *F*_peak_ results solely from the eccentric phase (i.e. force enhancement effects). At 60% activation, a relative increase of 20% in *F*_onset_ from SSC1 to SSC3, together with a relative increase of 5% in *F*_min_, contributed to a relative increase of 30% in *F*_peak_. At 20% activation, we observed the highest relative increase in *F*_onset_ (29%) and a relative increase in *F*_min_ (15%), with comparatively lower relative increases in *F*_peak_ (13%). This suggests that at low activation levels, changes in cross-bridge dynamics (e.g. increased number of cross-bridges), rather than titin-associated (force enhancement) effects during stretching, play a more dominant role in enhancing SSC performance across multiple cycles. At maximal activation, where all available cross-bridges are already bound, titin-associated effects appear to contribute more significantly to higher *F*_peak_ with increasing cycle number. This conclusion is also underpinned by the fact that shortening forces do not change substantially at maximal activation (Fig. 3 C, 100% activation). Based on this, we further speculate that an increase in force during shortening may partly result from a rise in the number of cross-bridges as cycle number increases. A corresponding trend is evident in our data from lower activation levels (Fig. 3 A & B, 20% and 60% activation).

### Comparison with literature

In general, changes in the force–time responses with increasing numbers of SSCs have been reported for consecutive SSCs with sustained activation (e.g. [3, 14, 44, 61]). In experiments with cat soleus muscle, consecutive SSCs (no delay) showed cumulative force depression effects, resulting in progressively decreased forces at the end of each shortening phase (*F*_min_) [61]. Hessel et al. [44] (no delay) and Campbell and Moss [14] (0.1-s delay) reported a decrease in peak force (*F*_peak_) with increasing SSC numbers. In contrast, Andersen et al. [5] demonstrated an almost 50% increase in rat EDL peak stretch force during 5 consecutive eccentric contractions when the muscle was deactivated and passively shortened in 5-s intervals between sequences; interestingly, no increase in consecutive peak stretch force was found in rat soleus muscle [5]. The results of this study showed an increase in *F*_peak_ and work during consecutive SSCs for all activations tested (Tables 1 and 2), while *F*_onset_ and *F*_min_ showed either increases or decreases depending on the activation level across consecutive SSCs. It is challenging to compare these results with the existing literature since other studies on repetitive SSCs have executed different protocols. Most studies have primarily focused on changes in force and initial stiffness during repetitive SSCs [13, 61], often without incorporating sufficiently long isometric hold phases between SSCs to allow force to return to a steady state. Consequently, in other studies, only the first SSC starts from an isometric steady state, while subsequent SSCs begin immediately or briefly after shortening, e.g. from lower force levels (see [3, 13, 44, 61]). Therefore, our *F*_onset_ cannot be directly compared, and the first SSC in the studies by Campbell and Moss [13], Lee et al. [61], Altman et al. [3], and Hessel et al. [44] must be excluded from comparisons due to the different starting conditions. Moreover, the aforementioned studies have not quantitatively compared *F*_onset_, *F*_peak_, and *F*_min_, or their changes throughout consecutive SSCs, although approximate data can be derived from the figures in these studies. Despite these figures often representing only exemplary data (*n* = 1), they illustrate trends useful for comparison. In summary, our results align with the literature showing that multiple SSCs can alter the force response [3, 14, 44].

### *F*_onset_ and *F*_min_

A methodologically similar study, in which multiple SSCs were performed starting from comparable isometric steady-state forces, was conducted by Campbell and Moss [14]. These authors investigated how the duration of an isometric hold phase between two SSCs (and thus also the time interval between two SSCs) affects the force response of a contracting rat soleus single muscle fibre, which was continuously activated using seven different activation levels (ranging from pCa 4.5 to pCa 6.5). Campbell and Moss [14] reported no increase in *F*_onset_ for maximum activation (pCa 4.5), which is consistent with our results (Table 1). Furthermore, they found an increase in *F*_onset_ for medium activations (pCa 5.8 and 6.2) and sufficiently long isometric contractions between cycles, which is also in agreement with our observations for 20% and 60% activation. The increased *F*_onset_ at medium activation could be a hint for several phenomena, including residual force enhancement [1], a higher rate of force redevelopment [4, 89], and/or an increased number of attached cross-bridges before the start of subsequent SSCs compared to the initial SSC [11]. Since the number of unbound cross-bridges is greater during submaximal contractions compared to maximal contractions [25, 85], potentially more cross-bridges would be available for additional binding induced by previous SSCs (for further explanation, see section ‘Potential mechanisms of performance enhancement’).

*F*_min_ showed a similar pattern to *F*_onset_ across different activation levels. Specifically, we observed an increase in *F*_min_ during consecutive SSCs at 20% and 60% activation, with increases of 15% and 5%, respectively, from SSC1 to SSC3. In contrast, *F*_min_ decreased by approximately 9% at 100% activation over the same interval. These findings are consistent with those of Lee et al. [61], who reported progressively smaller minimum forces achieved after each shortening phase in their triple SSC experiments on cat soleus muscle at 100% activation. Since both *F*_min_ and *F*_onset_ increase at activation levels of 20% and 60% but show no increase at 100% activation, it can be assumed that similar underlying processes could be responsible for the development of both force parameters over repeated cycles.

### *F*_peak_

We found an increase in *F*_peak_ with repetitive SSCs. In general, the results of Campbell and Moss [14] agree with our data. For long isometric contractions that allowed the fibre to reach a complete steady-state isometric force before the SSC, Campbell and Moss ([14], their Fig. 10) reported an increase in force at the end of the eccentric ramp from the first to the second SSC, except for the lowest activation level of about 10% (pCa 6.5). However, since Campbell and Moss [14] used higher stretch velocities of 0.1 *L*_0_/s (compared to 0.024 *L*_0_/s in our study, which corresponds to 1% *v*_max_), their experiments exhibited a clear muscle give ([14], their Figs. 4 & 5). Muscle give refers to a velocity-dependent phenomenon where the muscle force decreases during the eccentric phase due to the detachment of active cross-bridges [26, 103]. After an initial force peak at the start of the eccentric phase, muscle give leads to a loss of force, which manifests as a negative slope or force drop during further stretching. However, due to the slow SSC velocity tested (1% *v*_max_), this is not observed in our results (Fig. 1). As muscle give is induced by the detachment of active cross-bridges [26, 103], this phenomenon can affect force development and force enhancement during the SSC, as well as the transfer of SSC effects to subsequent cycles. This introduces challenges in comparing results across studies. Another set of experiments by Campbell and Moss [13], conducted at stretch velocities of 0.09 *L*_0_/s on permeabilised rat psoas fibres, included an isometric hold phase of 0.1 s between multiple SSCs. Their Fig. 1 also shows an increase in *F*_peak_ between the second and third SSC.

In concerning studies involving consecutive SSCs without delay between cycles, experiments on cat soleus muscles showed no change in *F*_peak_ with increasing cycle number [61] and a reduction in peak force during multiple SSCs in permeabilised mouse psoas fibres [44]. Since the latter experiments were conducted at longer sarcomere lengths on the descending limb of the force–length relation, there is a reduced number of cross-bridges. If force enhancement effects depend on the number of active cross-bridges [62, 63], the reported decrease in *F*_peak_ could additionally be due to a reduced contribution of force enhancement effects in repetitive SSCs, resulting from initial activations at longer muscle lengths on the descending limb of the force–length relation.

SSC velocity might have an impact on our results. A key study investigating the effects of contraction velocities during repetitive cyclic contractions was performed by Altman et al. [3] on maximally activated rabbit psoas fibres. They reported that peak force increased from cycle to cycle during slow SSC contractions (< 1 Hz cycling frequency), while it decreased with repetitive cycling at higher frequencies (> 1 Hz). As our SSCs were performed at very slow cycling frequencies (0.067 Hz), the results align with the observations from Altman et al. [3]. Further, a previous study [98] demonstrated that the SSC effect is velocity-dependent, showing increased force peaks, higher work output, and lower force minima at higher velocities. Based on these findings, it can be hypothesised that increasing SSC velocities under similar experimental conditions would amplify the effects on the tested parameters. However, the potential impact of velocity on consecutive SSCs remains speculative.

### Work

We found a mean increase in *Work*_SSC_ from SSC1 to SSC3 of about 61% across conditions (Table 2), which can be partly explained by increases in *F*_onset_, *F*_peak_, and* F*_min_. The work-loop method, introduced by Josephson [51], is often used to analyse the work performed by muscles during repetitive contractions. However, studies directly reporting muscle work output over multiple SSCs with continuous activation are sparse. Although there is no direct specification of work, Campbell and Moss [13] suggested from their Figs. 2 and 3 that in experiments on rabbit psoas, there were minimal changes in work per cycle. Their force–length curves for the repetitive cycles are approximately identical, except for the first cycle, which exhibits a clear muscle give compared to the subsequent cycles. The increase in the force response and performance from SSC to SSC depends on numerous parameters. For example, low stretch velocities, sufficient constant isometric activation between the SSCs, and work over the plateau region of the force–length curve appear to positively affect performance enhancement compared with the previous cycle in experiments with continuous muscle activation [5, 14]. *Work*_SHO_ showed no significant change over multiple SSCs at 100% activation. In contrast, *Work*_SHO_ increased significantly at both 20% and 60% activation. This increase aligns with the changes observed in *F*_onset_ and *F*_min_, indicating that SSC performance enhancing effects are more pronounced in submaximal activations, where additional cross-bridges can potentially be recruited with increasing cycle number. However, further experiments that isolate the role of titin and cross-bridges in different activation states are necessary to better understand the complex interaction of these individual factors. Specifically, experiments using titin-modified muscle preparations (e.g. [93]) could help to determine whether the potentiation effects at lower activation levels are primarily due to the recruitment of additional cross-bridges or if titin itself plays a significant role in the observed force enhancement.

### Potential mechanisms of performance enhancement

Force generation in muscle fibres during repeated SSCs is influenced by several structures whose properties change depending on previous contractions [36, 39, 90]. These modifications could help explain the observed improvements in performance.

Slow SSCs over the plateau region of the force–length relationship are associated with positive cumulative effects on muscle force [3], unlike the negative effects observed on the descending limb [44]. These findings suggest that the number of attached cross-bridges may affect the outcome and impact of cumulative effects during multiple SSCs. With the maximum number of available cross-bridges attached at the force plateau for isometric contraction, additional (former unavailable) cross-bridges in an ‘OFF’ state may be recruited into an ‘ON’ state during the stretching phase of the SSC, contributing to increased force production after the first SSC [31, 64]. Cross-bridge-based mechanisms can potentially be responsible for the increase in *F*_onset_, *F*_peak_, and *F*_min_*.* Muscle contractility depends not only on thin filament (actin) activation (via calcium binding to troponin C) but also on thick filament (myosin) activation [50, 64]. Myosin heads exist in a spectrum between two states: the OFF state, where they are docked against the thick filament backbone and unable to bind to actin, and the ON state, where they are free to interact with actin and generate force [41, 74]. Many authors described a ‘mechanosensing’ mechanism, where the mechanical load experienced by myosin during contraction facilitates the transition of more myosin heads from the OFF state into the ON state. This process increases the number of active cross-bridges (for a review, see [10]). Other mechanisms (e.g. higher Ca^2+^ and ADP concentrations, regulatory light chain phosphorylation) have also been proposed to cause cross-bridges in the OFF state to shift into the ON state [20, 72, 74]. High eccentric forces not only occur in actin and myosin but also in titin, where they trigger a cascade of changes [76]. It is proposed that mechanical load during contraction pulls on titin, which in turn interacts with myosin-binding protein-C [10, 41, 42]. Myosin-binding protein-C is thought to have a bridging function between actin and myosin, with conformational changes induced by titin pulling that may lead to a release of myosin heads from the OFF to the ON state [10, 38, 41, 64], which might contribute to an increased number of attached cross-bridges.

The high forces experienced by myosin and titin during the first SSC could lead to an increase in the number of cross-bridges in the ON state. The ON state could be maintained until the next SSC, as supported by data showing optimised force redevelopment after a SSC [89, 99]. The increased number of cross-bridges in the ON state would then contribute to higher forces in subsequent muscle actions. The increase in active cross-bridges causes additional stress on both myosin and titin, which could trigger a feedback loop, promoting the transition of even more cross-bridges into the ON state. Keeping the fibre activated, this process may persist into subsequent SSCs, amplifying force production with each cycle and contributing to the observed increase in force across consecutive SSCs. An increased number of active cross-bridges aligns with our observed increases in *F*_onset_, *F*_peak_, *F*_min_, and *Work*_SHO_ at 20% and 60% activations (see Tables 1 and 2).

In this context, one limitation of our study is the experimental temperature of 12 °C, which was selected to maintain fibre stability during prolonged activations and active lengthening. It has been shown that at this temperature, myosin ON/OFF states may not be as dynamically regulated as at physiological temperatures, with a greater proportion of myosin heads remaining in the ON state [15, 16, 91]. This could potentially influence cross-bridge cycling dynamics and the role of thick filament activation in force production. However, at 100% activation, the observed decrease in *F*_min_, along with the lack of increase in *F*_onset_, *and Work*_SHO_, suggests that an increase in the number of attached cross-bridges is unlikely at full muscle activation. Despite this, an increase in *F*_peak_ was still observed at 100% activation (Table 1), indicating that another mechanism may contribute to transferring performance enhancements to subsequent cycles.

While changes in force during consecutive SSCs can be attributed to variations in cross-bridge number, it is important to consider that cross-bridge kinetics also play a crucial role. Even at maximal activation, where most available cross-bridge sites are likely occupied, alterations in attachment/detachment rates could still influence force production. Additionally, force changes may not exclusively result from cross-bridge behaviour but also stem from alterations in force transmission within the sarcomere or the muscle fibre. Structural factors such as sarcomere compliance, filament extensibility, variations in myofilament lattice, or stretched intermyofilament bridge elements (e.g. titin, myosin-binding protein-C) could influence how force is transmitted within and between sarcomeres, thereby affecting the overall force output [43].

### Titin

The mechanism allowing to transfer performance enhancement even at maximum activation states could be related to the unique properties of titin. Supporting this idea, Altman et al. [3] demonstrated that the increase in peak forces during active muscle lengthening observed at slow contraction velocities in intact fibres was also present after treatment with a chemical cross-bridge inhibitor like Blebbistatin. This, in turn, suggests that non-cross-bridge structures also contribute to changes in force during consecutive SSCs.

Accumulating evidence suggests that the giant sarcomeric protein titin is a Ca^2+^-sensitive, non-cross-bridge viscoelastic element that functions as a tunable spring in active muscle [24, 40, 63, 76, 95]. Numerous recent studies support the hypothesis that titin’s N2A region is a signalling hub [76, 78]. Titin’s force generation is also modulated by calcium binding, altering its stiffness [52, 59]. Furthermore, it is supposed that titin binds to actin in the presence of Ca^2+^ [55], and the proposed binding sites include the PEVK region [67, 106] or areas in and around the N2A segment of titin [24, 80].

Although there is no study on titin contribution to performance enhancement during consecutive SSCs, the following studies suggested that an increase in titin stiffness during activation might be possible. In this context, using a Blebbistatin cross-bridge-block, Tomalka et al. [99] showed that non-cross-bridge structures contributed to faster and greater force redevelopment after a SSC compared with pure shortening contractions. This optimised state for force generation may also contribute to enhanced performance in a subsequent cycle, particularly if the muscle or fibre remains continuously activated. This effect could help explain the observed increase in *F*_peak_ across all levels of activation tested. Alternatively, several post-translational modifications of titin could contribute to an acute increase in titin stiffness during consecutive SSCs, enabling direct modulations of titin forces. These modifications include calcium ion binding and variations in Ca^2+^ sensitivity [102], chaperone binding [101], titin-actin interactions [106], and oxidation [2, 35]. Furthermore, experimental studies have shown that protein kinase phosphorylation [27, 45] can significantly alter the stiffness of the PEVK and N2B (in the heart) or N2A (in skeletal muscle) spring elements of titin. This enables a rapid adaptation of titin stiffness, which is crucial for adjusting cardiac output in response to haemodynamic stress (Frank-Starling mechanism) or for accommodating acute muscle actions such as eccentric contractions and SSCs [45]. Additionally, a study by Müller et al. [73] found a significant increase in titin phosphorylation in skeletal muscle after only 15 min of eccentric contractions on the vastus lateralis of the mouse, which was accompanied by a significant increase in titin stiffness. Similar results were reported by Rose and Hargreaves [84], who observed increased phosphorylation rates in skeletal muscle within a few minutes following acute exercise. Consequently, titin stiffness can be dynamically and acutely modulated through various post-translational modifications. Altered phosphorylation of the N2A and PEVK regions leads to an increase in titin stiffness [46], probably by strengthening intramolecular electrostatic bonds [66]. These modifications can occur under various physiological conditions (such as acute exercise loads within a few minutes or even seconds in permeabilised muscle preparations). Since the N2A and PEVK regions are also anchor points for signalling proteins, these regions likely have stretch-induced effects on the binding of signalling molecules, thereby contributing to tension- and phosphorylation-dependent mechanosignalling [60]. An increase in titin stiffness over several consecutive SSCs will induce an increase in *F*_peak_ [83], which we were able to observe for all activation levels.

## Conclusion

This study demonstrated that when a muscle fibre remains active during consecutive SSCs with an isometric hold phase between cycles, both force production and mechanical work increase in the subsequent SSCs. The performance enhancements observed with each SSC may involve changes at both the cross-bridge and non-cross-bridge levels. The level of activation plays a key role in determining the contribution of different mechanisms to performance enhancement, with cross-bridge enhancements dominating at low to medium activation levels, while titin-associated-stretch-enhancing effects become more pronounced at high activation levels. Although these findings align with existing literature, further research is needed to clarify how factors such as stretch velocity, stretch amplitude, muscle activation, and time intervals between cycles influence these mechanisms and their contribution to performance enhancement.

## Supplementary Information

Below is the link to the electronic supplementary material.

ESM 1 (DOCX 1.57 MB)

## Data Availability

Data will be made public upon request.

## References

[1] Abbott BC, Aubert XM (1952) The force exerted by active striated muscle during and after change of length. J Physiol 117:77. 10.1113/jphysiol.1952.sp004733 14946730 PMC1392571

[2] Alegre-Cebollada J, Kosuri P, Giganti D, Eckels E, Rivas-Pardo JA, Hamdani N, Warren CM, Solaro RJ, Linke WA, Fernández JM (2014) S-Glutathionylation of cryptic cysteines enhances titin elasticity by blocking protein folding. Cell 156:1235–1246. 10.1016/j.cell.2014.01.056 24630725 10.1016/j.cell.2014.01.056 PMC3989842

[3] Altman D, Minozzo FC, Rassier DE (2015) Thixotropy and rheopexy of muscle fibers probed using sinusoidal oscillations. PLoS ONE 10(4):e0121726. 10.1371/journal.pone.0121726 10.1371/journal.pone.0121726 PMC4400131 25880774

[4] Ames SR, Joumaa V, Herzog W (2022) Effect of active shortening and stretching on the rate of force re-development in rabbit psoas muscle fibres. J Exp Biol 225(22):jeb244703. 10.1242/jeb.244703 10.1242/jeb.244703 36268629

[5] Andersen OE, Kristensen AM, Nielsen OB, Overgaard K (2023) Force potentiation during eccentric contractions in rat skeletal muscle. J Appl Physiol 134:777–785. 10.1152/japplphysiol.00676.2022 36759160 10.1152/japplphysiol.00676.2022

[6] Araz M, Weidner S, Izzi F, Badri-Spröwitz A, Siebert T, Haeufle DFB (2023) Muscle preflex response to perturbations in locomotion: In vitro experiments and simulations with realistic boundary conditions. Front Bioeng Biotechnol 11:1150170. 10.3389/fbioe.2023.1150170 10.3389/fbioe.2023.1150170 PMC10194126 37214305

[7] Berg HE, Eiken O (1999) Muscle control in elite alpine skiing. Med Sci Sports Exerc 31:1065–1067. 10.1097/00005768-199907000-00022 10416571 10.1097/00005768-199907000-00022

[8] Bobbert MF, Casius LJR (2005) Is the effect of a countermovement on jump height due to active state development? Med Sci Sports Exerc 37:440–446. 10.1249/01.MSS.0000155389.34538.97 15741843 10.1249/01.mss.0000155389.34538.97

[9] Bosco C, Montanari G, Ribacchi R, Giovenali P, Latteri F, Iachelli G, Faina M, Colli R, Dal Monte A, La Rosa M, Cortili G, Saibene F (1987) Relationship between the efficiency of muscular work during jumping and the energetics of running. Eur J Appl Physiol Occup Physiol 56:138–143. 10.1007/BF00640636 3569218 10.1007/BF00640636

[10] Brunello E, Fusi L (2024) Regulating striated muscle contraction: through thick and thin. Annu Rev Physiol 86:255–275. 10.1146/annurev-physiol-042222-022728 37931167 10.1146/annurev-physiol-042222-022728

[11] Brunello E, Marcucci L, Irving M, Fusi L (2023) Activation of skeletal muscle is controlled by a dual-filament mechano-sensing mechanism. Proc Natl Acad Sci 120(22). 10.1073/pnas.2302837120 10.1073/pnas.2302837120 PMC10235942 37216507

[12] Burkholder TJ, Lieber RL (2001) Sarcomere length operating range of vertebrate muscles during movement. J Exp Biol 204:1529–1536. 10.1242/jeb.204.9.1529 11296141 10.1242/jeb.204.9.1529

[13] Campbell KS, Moss RL (2000) A thixotropic effect in contracting rabbit psoas muscle: prior movement reduces the initial tension response to stretch. J Physiol 525:531–548. 10.1111/j.1469-7793.2000.00531.x 10835052 10.1111/j.1469-7793.2000.00531.x PMC2269955

[14] Campbell KS, Moss RL (2002) History-dependent mechanical properties of permeabilized rat soleus muscle fibers. Biophys J 82:929–943. 10.1016/S0006-3495(02)75454-4 11806934 10.1016/S0006-3495(02)75454-4 PMC1301901

[15] Caremani M, Brunello E, Linari M, Fusi L, Irving TC, Gore D, Piazzesi G, Irving M, Lombardi V, Reconditi M (2019) Low temperature traps myosin motors of mammalian muscle in a refractory state that prevents activation. J Gen Physiol 151:1272–1286. 10.1085/jgp.201912424 31554652 10.1085/jgp.201912424 PMC6829559

[16] Caremani M, Fusi L, Linari M, Reconditi M, Piazzesi G, Irving TC, Narayanan T, Irving M, Lombardi V, Brunello E (2021) Dependence of thick filament structure in relaxed mammalian skeletal muscle on temperature and interfilament spacing. J Gen Physiol 153(3):e202012713. 10.1085/jgp.202012713 10.1085/jgp.202012713 PMC7802359 33416833

[17] Cavagna GA, Dusman B, Margaria R (1968) Positive work done by a previously stretched muscle. J Appl Physiol 24:21–32. 10.1152/jappl.1968.24.1.21 5635766 10.1152/jappl.1968.24.1.21

[18] Chapman N, Whitting J, Broadbent S, Crowley-McHattan Z, Meir R (2018) Residual force enhancement in humans: a systematic review. J Appl Biomech 34:240–248. 10.1123/jab.2017-0234 29364041 10.1123/jab.2017-0234

[19] Clarys JP, Alewaeters K, Zinzen E (2001) The influence of geographic variations on the muscular activity in selected sports movements. J Electromyogr Kinesiol 11:451–457. 10.1016/S1050-6411(01)00020-7 11738957 10.1016/s1050-6411(01)00020-7

[20] Craig R, Padrón R (2021) Structural basis of the super- and hyper-relaxed states of myosin II. J Gen Physiol 154(1). 10.1085/jgp.202113012 10.1085/jgp.202113012 PMC8669498 34889960

[21] Curtin NA, Diack RA, West TG, Wilson AM, Woledge RC (2015) Skinned fibres produce the same power and force as intact fibre bundles from muscle of wild rabbits. J Exp Biol. 10.1242/jeb.121897 26206354 10.1242/jeb.121897

[22] Degens H, Yu F, Li X, Larsson L (1998) Effects of age and gender on shortening velocity and myosin isoforms in single rat muscle fibres. Acta Physiol Scand 163:33–40. 10.1046/j.1365-201x.1998.00329.x 9648621 10.1046/j.1365-201x.1998.00329.x

[23] Dietz V, Schmidtbleicher D, Noth J (1979) Neuronal mechanisms of human locomotion. J Neurophysiol 42:1212–1222. 10.1152/jn.1979.42.5.1212 490196 10.1152/jn.1979.42.5.1212

[24] Dutta S, Tsiros C, Sundar SL, Athar H, Moore J, Nelson B, Gage MJ, Nishikawa K (2018) Calcium increases titin N2A binding to F-actin and regulated thin filaments. Sci Rep 8:14575. 10.1038/s41598-018-32952-8 30275509 10.1038/s41598-018-32952-8 PMC6167357

[25] Ebashi S, Endo M (1968) Calcium and muscle contraction. Prog Biophys Mol Biol 18:123–183. 10.1016/0079-6107(68)90023-0 4894870 10.1016/0079-6107(68)90023-0

[26] Flitney FW, Hirst DG (1978) Cross-bridge detachment and sarcomere “give” during stretch of active frog’s muscle. J Physiol 276:449–465. 10.1113/jphysiol.1978.sp012246 306433 10.1113/jphysiol.1978.sp012246 PMC1282437

[27] Freundt JK, Linke WA (2019) Titin as a force-generating muscle protein under regulatory control. J Appl Physiol 126:1474–1482. 10.1152/japplphysiol.00865.2018 30521425 10.1152/japplphysiol.00865.2018

[28] Fukutani A, Herzog W (2019) Influence of stretch magnitude on the stretch-shortening cycle in skinned fibres. J Exp Biol. 10.1242/jeb.206557 31171600 10.1242/jeb.206557

[29] Fukutani A, Joumaa V, Herzog W (2017) Influence of residual force enhancement and elongation of attached cross-bridges on stretch-shortening cycle in skinned muscle fibers. Physiol Rep 5(22):e13477. 10.14814/phy2.13477 10.14814/phy2.13477 PMC5704075 29180479

[30] Fukutani A, Leonard T, Herzog W (2019) Does stretching velocity affect residual force enhancement? J Biomech 89:143–147. 10.1016/j.jbiomech.2019.04.033 31060810 10.1016/j.jbiomech.2019.04.033

[31] Fusi L, Brunello E, Yan Z, Irving M (2016) Thick filament mechano-sensing is a calcium-independent regulatory mechanism in skeletal muscle. Nat Commun 7:13281. 10.1038/ncomms13281 10.1038/ncomms13281 PMC5095582 27796302

[32] Goecking T, Holzer D, Hahn D, Siebert T, Seiberl W (2024) Unlocking the benefit of active stretch: the eccentric muscle action not the preload maximizes muscle-tendon unit stretch-shortening cycle performance. J Appl Physiol. 10.1152/japplphysiol.00809.2023 38932683 10.1152/japplphysiol.00809.2023

[33] Gordon AM, Huxley AF, Julian FJ (1966) The variation in isometric tension with sarcomere length in vertebrate muscle fibres. J Physiol 184:170–192. 10.1113/jphysiol.1966.sp007909 5921536 10.1113/jphysiol.1966.sp007909 PMC1357553

[34] Gregor RJ, Roy RR, Whiting WC, Lovely RG, Hodgson JA, Edgerton VR (1988) Mechanical output of the cat soleus during treadmill locomotion: In vivo vs in situ characteristics. J Biomech 21:721–732. 10.1016/0021-9290(88)90281-3 3182876 10.1016/0021-9290(88)90281-3

[35] Grützner A, Garcia-Manyes S, Kötter S, Badilla CL, Fernandez JM, Linke WA (2009) Modulation of titin-based stiffness by disulfide bonding in the cardiac titin N2-B unique sequence. Biophys J 97:825–834. 10.1016/j.bpj.2009.05.037 19651040 10.1016/j.bpj.2009.05.037 PMC2718153

[36] Hahn D, Han S, Joumaa V (2023) The history-dependent features of muscle force production: a challenge to the cross-bridge theory and their functional implications. J Biomech 152:111579. 10.1016/j.jbiomech.2023.111579 37054597 10.1016/j.jbiomech.2023.111579

[37] Hahn D, Riedel TN (2018) Residual force enhancement contributes to increased performance during stretch-shortening cycles of human plantar flexor muscles in vivo. J Biomech 77:190–193. 10.1016/j.jbiomech.2018.06.003 29935734 10.1016/j.jbiomech.2018.06.003

[38] Harris SP (2021) Making waves: a proposed new role for myosin-binding protein C in regulating oscillatory contractions in vertebrate striated muscle. J Gen Physiol 153(3):e202012729. 10.1085/jgp.202012729 10.1085/jgp.202012729 PMC7721898 33275758

[39] Herzog W (1998) History dependence of force production in skeletal muscle: a proposal for mechanisms. J Electromyogr Kinesiol 8:111–117. 10.1016/S1050-6411(97)00027-8 9680951

[40] Herzog W (2019) Passive force enhancement in striated muscle. J Appl Physiol 126:1782–1789. 10.1152/japplphysiol.00676.2018 31070958 10.1152/japplphysiol.00676.2018 PMC6620658

[41] Hessel AL, Engels NM, Kuehn MN, Nissen D, Sadler RL, Ma W, Irving TC, Linke WA, Harris SP (2024) Myosin-binding protein C regulates the sarcomere lattice and stabilizes the OFF states of myosin heads. Nat Commun 15(1):2628. 10.1038/s41467-024-46957-7 10.1038/s41467-024-46957-7 PMC10960836 38521794

[42] Hessel AL, Kuehn MN, Han S-W, Ma W, Irving TC, Momb BA, Song T, Sadayappan S, Linke WA, Palmer BM (2024) Fast myosin binding protein C knockout in skeletal muscle alters length-dependent activation and myofilament structure. Commun Biol 7:648. 10.1038/s42003-024-06265-8 38802450 10.1038/s42003-024-06265-8 PMC11130249

[43] Hessel AL, Kuehn MN, Palmer BM, Nissen D, Mishra D, Joumaa V, Freundt JK, Ma W, Nishikawa KC, Irving TC, Linke WA (2024) The distinctive mechanical and structural signatures of residual force enhancement in myofibers. Proc Natl Acad Sci USA 121(52):e2413883121. 10.1073/pnas.2413883121 10.1073/pnas.2413883121 PMC11670058 39680764

[44] Hessel AL, Monroy JA, Nishikawa KC (2021) Non-cross bridge viscoelastic elements contribute to muscle force and work during stretch-shortening cycles: evidence from whole muscles and permeabilized Fibers. Front Physiol 12:648019. 10.3389/fphys.2021.648019 10.3389/fphys.2021.648019 PMC8039322 33854441

[45] Hidalgo C, Granzier H (2013) Tuning the molecular giant titin through phosphorylation: role in health and disease. Trends Cardiovasc Med 23:165–171. 10.1016/j.tcm.2012.10.005 23295080 10.1016/j.tcm.2012.10.005 PMC3622841

[46] Hidalgo C, Hudson B, Bogomolovas J, Zhu Y, Anderson B, Greaser M, Labeit S, Granzier H (2009) PKC phosphorylation of titin’s PEVK element. Circ Res 105:631–638. 10.1161/CIRCRESAHA.109.198465 19679839 10.1161/CIRCRESAHA.109.198465 PMC2764991

[47] Holt NC, Roberts TJ, Askew GN (2014) The energetic benefits of tendon springs in running: is the reduction of muscle work important? J Exp Biol. 10.1242/jeb.112813 25394624 10.1242/jeb.112813 PMC4375839

[48] Huijing PA (1991) Elastic potential of muscle. In: Komi PV (ed) Strength and power in sport. Blackwell, Oxford, pp 151–168

[49] Huxley AF (1957) Muscle structure and theories of contraction. Prog Biophys Biophys Chem 7:255–318. 10.1016/S0096-4174(18)30128-8 13485191

[50] Irving M (2017) Regulation of contraction by the thick filaments in skeletal muscle. Biophys J 113:2579–2594. 10.1016/j.bpj.2017.09.037 29262355 10.1016/j.bpj.2017.09.037 PMC5770512

[51] Josephson RK (1985) Mechanical power output from striated muscle during cyclic contraction. J Exp Biol 114:493–512. 10.1242/jeb.114.1.493

[52] Joumaa V, Rassier DE, Leonard TR, Herzog W (2008) The origin of passive force enhancement in skeletal muscle. Am J Physiol Cell Physiol 294:C74–C78. 10.1152/ajpcell.00218.2007 17928540 10.1152/ajpcell.00218.2007

[53] Kawakami Y, Muraoka T, Ito S, Kanehisa H, Fukunaga T (2002) In vivo muscle fibre behaviour during counter-movement exercise in humans reveals a significant role for tendon elasticity. J Physiol 540:635–646. 10.1113/jphysiol.2001.013459 11956349 10.1113/jphysiol.2001.013459 PMC2290252

[54] Kellermayer MSZ, Granzier HL (1996) Calcium-dependent inhibition of in vitro thin-filament motility by native titin. FEBS Lett 380:281–286. 10.1016/0014-5793(96)00055-5 8601441 10.1016/0014-5793(96)00055-5

[55] Kellermayer MS, Granzier HL (1996) Elastic properties of single titin molecules made visible through fluorescent F-actin binding. Biochem Biophys Res Commun 221(3):491–497. 10.1006/bbrc.1996.0624 10.1006/bbrc.1996.0624 8629989

[56] Komi PV (2000) Stretch-shortening cycle: a powerful model to study normal and fatigued muscle. J Biomech 33:1197–1206. 10.1016/S0021-9290(00)00064-6 10899328 10.1016/s0021-9290(00)00064-6

[57] Komi PV (2003) Stretch-shortening cycle. In: Komi PV (ed) Strength and power in sport. 10.1002/9780470757215.ch10

[58] Labeit D, Watanabe K, Witt C, Fujita H, Wu Y, Lahmers S, Funck T, Labeit S, Granzier H (2003) Calcium-dependent molecular spring elements in the giant protein titin. Proc Natl Acad Sci 100:13716–13721. 10.1073/pnas.2235652100 14593205 10.1073/pnas.2235652100 PMC263879

[59] Labeit S, Kolmerer B, Linke WA (1997) The giant protein titin. Circ Res 80:290–294. 10.1161/01.RES.80.2.290 9012751 10.1161/01.res.80.2.290

[60] Lanzicher T, Zhou T, Saripalli C, Keschrumrus V, Smith Iii JE, Mayans O, Sbaizero O, Granzier H (2020) Single-molecule force spectroscopy on the N2A element of titin: effects of phosphorylation and CARP. Front Physiol 11:173. 10.3389/fphys.2020.00173 10.3389/fphys.2020.00173 PMC7093598 32256378

[61] Lee H-D, Herzog W, Leonard T (2001) Effects of cyclic changes in muscle length on force production in in-situ cat soleus. J Biomech 34:979–987. 10.1016/S0021-9290(01)00077-X 11448689 10.1016/s0021-9290(01)00077-x

[62] Leonard TR, DuVall M, Herzog W (2010) Force enhancement following stretch in a single sarcomere. Am J Physiol Cell Physiol 299:C1398–C1401. 10.1152/ajpcell.00222.2010 20844251 10.1152/ajpcell.00222.2010

[63] Leonard TR, Herzog W (2010) Regulation of muscle force in the absence of actin-myosin-based cross-bridge interaction. Am J Physiol Cell Physiol 299:14–20. 10.1152/ajpcell.00049.2010 10.1152/ajpcell.00049.2010 20357181

[64] Linari M, Brunello E, Reconditi M, Fusi L, Caremani M, Narayanan T, Piazzesi G, Lombardi V, Irving M (2015) Force generation by skeletal muscle is controlled by mechanosensing in myosin filaments. Nature 528:276–279. 10.1038/nature15727 26560032 10.1038/nature15727

[65] Linari M, Caremani M, Piperio C, Brandt P, Lombardi V (2007) Stiffness and fraction of myosin motors responsible for active force in permeabilized muscle fibers from rabbit psoas. Biophys J 92:2476–2490. 10.1529/biophysj.106.099549 17237201 10.1529/biophysj.106.099549 PMC1864836

[66] Linke WA (2023) Stretching the story of titin and muscle function. J Biomech 152:111553. 10.1016/j.jbiomech.2023.111553 36989971 10.1016/j.jbiomech.2023.111553

[67] Linke WA, Kulke M, Li H, Fujita-Becker S, Neagoe C, Manstein DJ, Gautel M, Fernandez JM (2002) PEVK domain of titin: an entropic spring with actin-binding properties. J Struct Biol 137:194–205. 10.1006/jsbi.2002.4468 12064946 10.1006/jsbi.2002.4468

[68] Marcucci L (2023) Muscle mechanics and thick filament activation: an emerging two-way interaction for the vertebrate striated muscle fine regulation. Int J Mol Sci 24:6265. 10.3390/ijms24076265 37047237 10.3390/ijms24076265 PMC10094676

[69] Maréchal G, Plaghki L (1979) The deficit of the isometric tetanic tension redeveloped after a release of frog muscle at a constant velocity. J Gen Physiol 73:453–467. 10.1085/jgp.73.4.453 312915 10.1085/jgp.73.4.453 PMC2215170

[70] Ma W, McMillen TS, Childers MC, Gong H, Regnier M, Irving T (2023) Structural OFF/ON transitions of myosin in relaxed porcine myocardium predict calcium-activated force. Proc Natl Acad Sci USA 120(5):e2207615120. 10.1073/pnas.2207615120 10.1073/pnas.2207615120 PMC9945958 36696446

[71] Meijer K (2002) History dependence of force production in submaximal stimulated rat medial gastrocnemius muscle. J Electromyogr Kinesiol 12:463–470. 10.1016/S1050-6411(02)00040-8 12435543 10.1016/s1050-6411(02)00040-8

[72] Mohran S, Kooiker K, Mahoney-Schaefer M, Mandrycky C, Kao K, Tu AY, Freeman J, Moussavi-Harami F, Geeves M, Regnier M (2024) The biochemically defined super relaxed state of myosin-A paradox. J Biol Chem 300(1):105565. 10.1016/j.jbc.2023.105565 10.1016/j.jbc.2023.105565 PMC10819765 38103642

[73] Müller AE, Kreiner M, Kötter S, Lassak P, Bloch W, Suhr F, Krüger M (2014) Acute exercise modifies titin phosphorylation and increases cardiac myofilament stiffness. Front Physiol 5:449. 10.3389/fphys.2014.00449 10.3389/fphys.2014.00449 PMC4238368 25477822

[74] Nag S, Trivedi DV (2021) To lie or not to lie: super-relaxing with myosins. Elife 10:1–21 10.7554/eLife.63703 PMC7875563 33565963

[75] Nicol C, Avela J, Komi PV (2006) The stretch-shortening cycle. Sports Med 36:977–999. 10.2165/00007256-200636110-00004 17052133 10.2165/00007256-200636110-00004

[76] Nishikawa K (2020) Titin: a tunable spring in active muscle. Physiology 35:209–217. 10.1152/physiol.00036.2019 32293234 10.1152/physiol.00036.2019

[77] Nishikawa KC, Monroy JA, Uyeno TE, Yeo SH, Pai DK, Lindstedt SL (2012) Is titin a ‘winding filament’? A new twist on muscle contraction. Proceedings of the Royal Society B: Biological Sciences 279:981–990. 10.1098/rspb.2011.1304 10.1098/rspb.2011.1304 PMC3259925 21900329

[78] Nishikawa K, Lindstedt SL, Hessel A, Mishra D (2020) N2A titin: signaling hub and mechanical switch in skeletal muscle. Int J Mol Sci 21:3974 32492876 10.3390/ijms21113974 PMC7312179

[79] Oskouei AE, Herzog W (2009) Activation-induced force enhancement in human adductor pollicis. J Electromyogr Kinesiol 19:821–828. 10.1016/j.jelekin.2008.02.009 18430589 10.1016/j.jelekin.2008.02.009

[80] Powers K, Nishikawa K, Joumaa V, Herzog W (2016) Decreased force enhancement in skeletal muscle sarcomeres with a deletion in titin. J Exp Biol. 10.1242/jeb.132027 26944495 10.1242/jeb.132027

[81] Ranatunga KW (1982) Temperature-dependence of shortening velocity and rate of isometric tension development in rat skeletal muscle. J Physiol 329:465–483. 10.1113/jphysiol.1982.sp014314 7143257 10.1113/jphysiol.1982.sp014314 PMC1224791

[82] Ranatunga KW (1984) The force-velocity relation of rat fast- and slow-twitch muscles examined at different temperatures. J Physiol 351:517–529. 10.1113/jphysiol.1984.sp015260 6747875 10.1113/jphysiol.1984.sp015260 PMC1193132

[83] Rode C, Siebert T, Blickhan R (2009) Titin-induced force enhancement and force depression: a ‘sticky-spring’ mechanism in muscle contractions? J Theor Biol 259:350–360. 10.1016/j.jtbi.2009.03.015 19306884 10.1016/j.jtbi.2009.03.015

[84] Rose AJ, Hargreaves M (2003) Exercise Increases Ca2+–calmodulin-dependent protein kinase II activity in human skeletal muscle. J Physiol 553:303–309. 10.1113/jphysiol.2003.054171 14565989 10.1113/jphysiol.2003.054171 PMC2343484

[85] Rüegg JC (1992) Calcium in muscle contraction. Springer, Berlin Heidelberg

[86] Schappacher-Tilp G, Leonard T, Desch G, Herzog W (2015) A novel three-filament model of force generation in eccentric contraction of skeletal muscles. PLoS ONE 10:e0117634. 10.1371/journal.pone.0117634 25816319 10.1371/journal.pone.0117634 PMC4376863

[87] van Schenau GJ, Bobbert MFI, de Haan A (1997) Mechanics and energetics of the stretch-shortening cycle: a stimulating discussion. J Appl Biomech 13:484–496

[88] Seiberl W, Hahn D, Power GA, Fletcher JR, Siebert T (2021) Editorial: the stretch-shortening cycle of active muscle and muscle-tendon complex: what, why and how it increases muscle performance?. Front Physiol 12:693141. 10.3389/fphys.2021.693141 10.3389/fphys.2021.693141 PMC8173190 34093246

[89] Seiberl W, Power GA, Herzog W, Hahn D (2015) The stretch-shortening cycle (SSC) revisited: residual force enhancement contributes to increased performance during fast SSCs of human m adductor pollicis. Physiol Rep 3:e12401. 10.14814/phy2.12401 25975646 10.14814/phy2.12401 PMC4463830

[90] Siebert T, Screen HR, Rode C (2020) Computational modelling of muscle, tendon, and ligaments biomechanics. In: Elsevier eBooks (pp 155–186). 10.1016/b978-0-12-819531-4.00008-0

[91] Squire JM, Knupp C (2021) Analysis methods and quality criteria for investigating muscle physiology using x-ray diffraction. J Gen Physiol 153(10):e202012778. 10.1085/jgp.202012778 10.1085/jgp.202012778 PMC8348228 34351359

[92] Stephenson DG, Williams DA (1982) Effects of sarcomere length on the force—pCa relation in fast- and slow-twitch skinned muscle fibres from the rat. J Physiol 333:637–653. 10.1113/jphysiol.1982.sp014473 7182478 10.1113/jphysiol.1982.sp014473 PMC1197268

[93] Tahir U, Monroy JA, Rice NA, Nishikawa KC (2020) Effects of a titin mutation on force enhancement and force depression in mouse soleus muscles. J Exp Biol 223(Pt 2):jeb197038. 10.1242/jeb.197038 10.1242/jeb.197038 31862847

[94] Talbot JA, Morgan DL (1996) Quantitative analysis of sarcomere non-uniformities in active muscle following a stretch. J Muscle Res Cell Motil 17:261–268. 10.1007/BF00124247 8793727 10.1007/BF00124247

[95] Tomalka A (2023) Eccentric muscle contractions: from single muscle fibre to whole muscle mechanics. Pflugers Arch 475:421–435. 10.1007/s00424-023-02794-z 36790515 10.1007/s00424-023-02794-z PMC10011336

[96] Tomalka A, Rode C, Schumacher J, Siebert T (2017) The active force–length relationship is invisible during extensive eccentric contractions in skinned skeletal muscle fibres. Proc Royal Soc B: Biol Sci 284:20162497. 10.1098/rspb.2016.2497 10.1098/rspb.2016.2497 PMC5443931 28469023

[97] Tomalka A, Weidner S, Hahn D, Seiberl W, Siebert T (2020) Cross-bridges and sarcomeric non-cross-bridge structures contribute to increased work in stretch-shortening cycles. Front Physiol 11:921. 10.3389/fphys.2020.00921 10.3389/fphys.2020.00921 PMC7399218 32848862

[98] Tomalka A, Weidner S, Hahn D, Seiberl W, Siebert T (2021) Power amplification increases with contraction velocity during stretch-shortening cycles of skinned muscle fibers. Front Physiol 12:644981. 10.3389/fphys.2021.644981 10.3389/fphys.2021.644981 PMC8044407 33868012

[99] Tomalka A, Weidner S, Hahn D, Seiberl W, Siebert T (2024) Force re-development after shortening reveals a role for titin in stretch–shortening performance enhancement in skinned muscle fibres. J Exp Biol 227(17):jeb247377. 10.1242/jeb.247377.10.1242/jeb.247377 39119673 10.1242/jeb.247377

[100] Turner AN, Jeffreys I (2010) The stretch-shortening cycle: proposed mechanisms and methods for enhancement. Strength Cond J 32:87–99. 10.1519/SSC.0b013e3181e928f9

[101] Unger A, Beckendorf L, Böhme P, Kley R, von Frieling-Salewsky M, Lochmüller H, Schröder R, Fürst DO, Vorgerd M, Linke WA (2017) Translocation of molecular chaperones to the titin springs is common in skeletal myopathy patients and affects sarcomere function. Acta Neuropathol Commun 5:72. 10.1186/s40478-017-0474-0 28915917 10.1186/s40478-017-0474-0 PMC5603016

[102] Vandenboom R, Gittings W, Smith IC, Grange RW, Stull JT (2013) Myosin phosphorylation and force potentiation in skeletal muscle: evidence from animal models. J Muscle Res Cell Motil 34:317–332. 10.1007/s10974-013-9363-8 24162313 10.1007/s10974-013-9363-8

[103] Weidner S, Tomalka A, Rode C, Siebert T (2022) How velocity impacts eccentric force generation of fully activated skinned skeletal muscle fibers in long stretches. J Appl Physiol 133:223–233. 10.1152/japplphysiol.00735.2021 35652830 10.1152/japplphysiol.00735.2021

[104] Weidner S, Tomalka A, Rode C, Siebert T (2024) Impact of lengthening velocity on the generation of eccentric force by slow-twitch muscle fibers in long stretches. Pflugers Arch. 10.1007/s00424-024-02991-4 39043889 10.1007/s00424-024-02991-4 PMC11381483

[105] Wilk KE, Voight ML, Keirns MA, Gambetta V, Andrews JR, Dillman CJ (1993) Stretch-shortening drills for the upper extremities: theory and clinical application. J Orthop Sports Phys Ther 17:225–239. 10.2519/jospt.1993.17.5.225 8343780 10.2519/jospt.1993.17.5.225

[106] Yamasaki R, Berri M, Wu Y, Trombitás K, McNabb M, Kellermayer MSZ, Witt C, Labeit D, Labeit S, Greaser M, Granzier H (2001) Titin–actin interaction in mouse myocardium: passive tension modulation and its regulation by calcium/S100A1. Biophys J 81:2297–2313. 10.1016/S0006-3495(01)75876-6 11566799 10.1016/S0006-3495(01)75876-6 PMC1301700

