# Supplementary material for: Consecutive SSCs increase the SSC effect in skinned rat muscle fibres
Source: Pflugers Arch. 2025 May 8;477(6):873–88. doi: 10.1007/s00424-025-03088-2 (PMC12092553; doi:10.1007/s00424-025-03088-2)
Supplement: Supplementary file 1 — (DOCX 1.57 MB) [file 424_2025_3088_MOESM1_ESM.docx]

**Supplementary information**


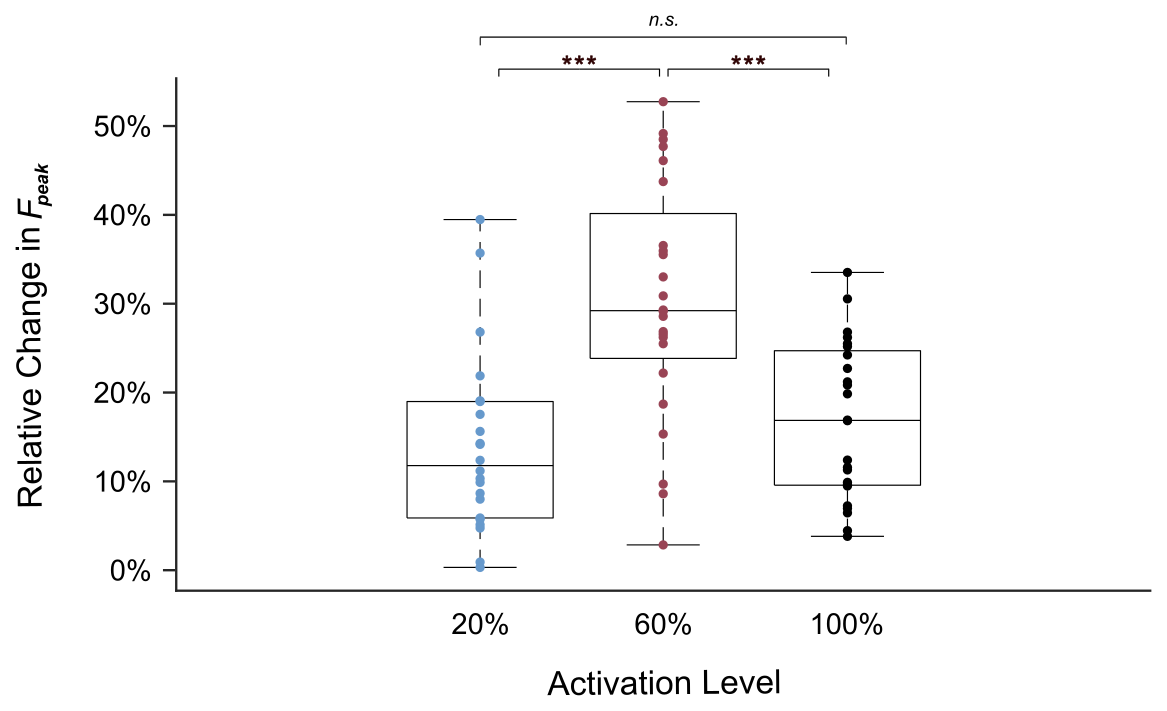


Figure S1. Degree of change in F_peak_ depending on activation. Repeated Measures ANOVA was performed to assess differences. ANOVA revealed that there is a difference [F(2, 42) = 24.160, p<0.001]. Post hoc tests showed a difference between the relative change in F_peak_ for 20% and 60% activation (p<0.001; ***), and for 60% and 100% activation (p<0.001; ***) but no difference for 20% and 100% activation (p=0.246; n.s).


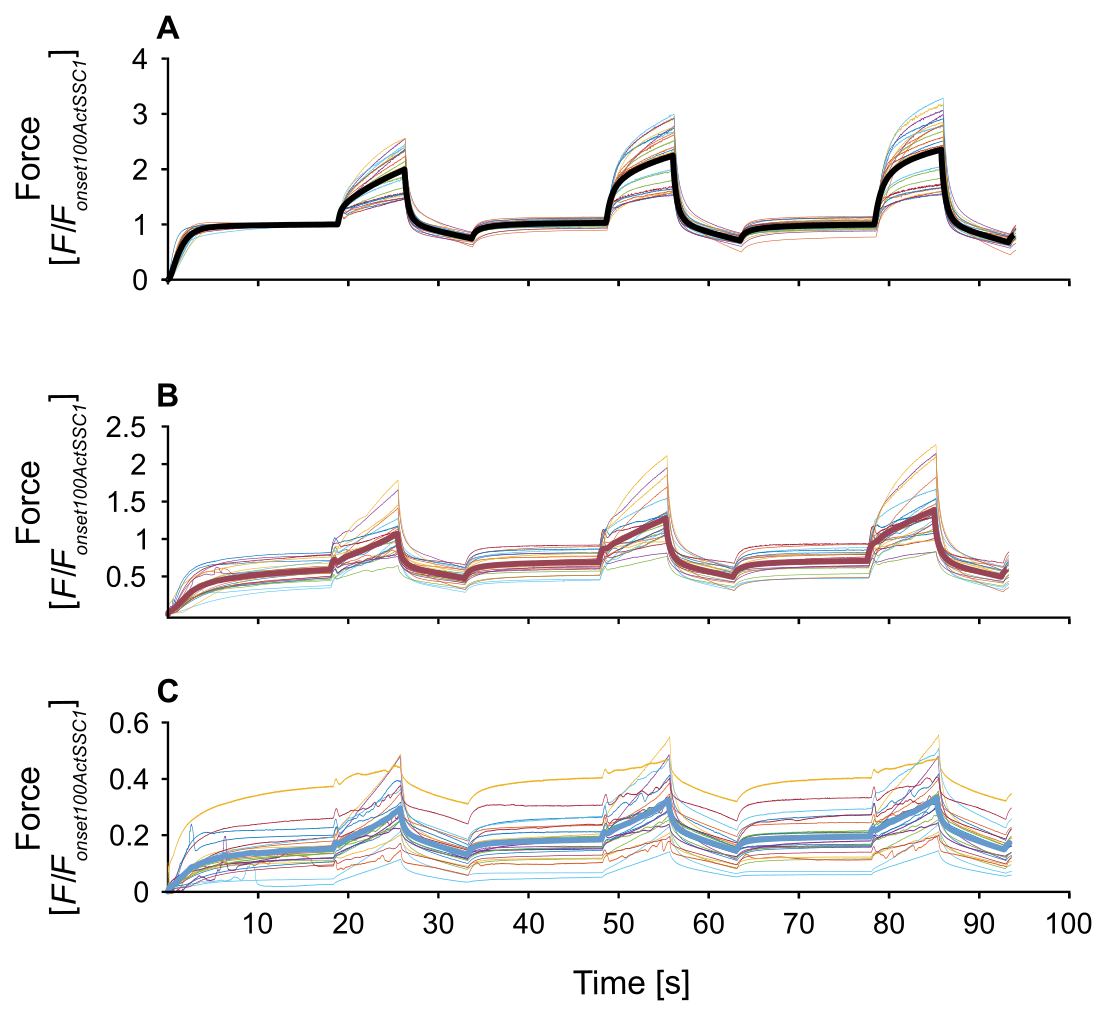


Figure S2. Presentation of all individual data (force-time traces) relative to the force at 100% activation immediately before SSC1 (F_onsetSSC1_). Each subplot contains 24 individual data points (except for 20% activation, where only 22 fibres were analysed). The bold lines represent the mean values. Note the differently scaled y-axis for each subplot. **(A)** shows the activation level of 100%, **(B)** the activation level of 60%, and **(C)** the activation level of 20%.


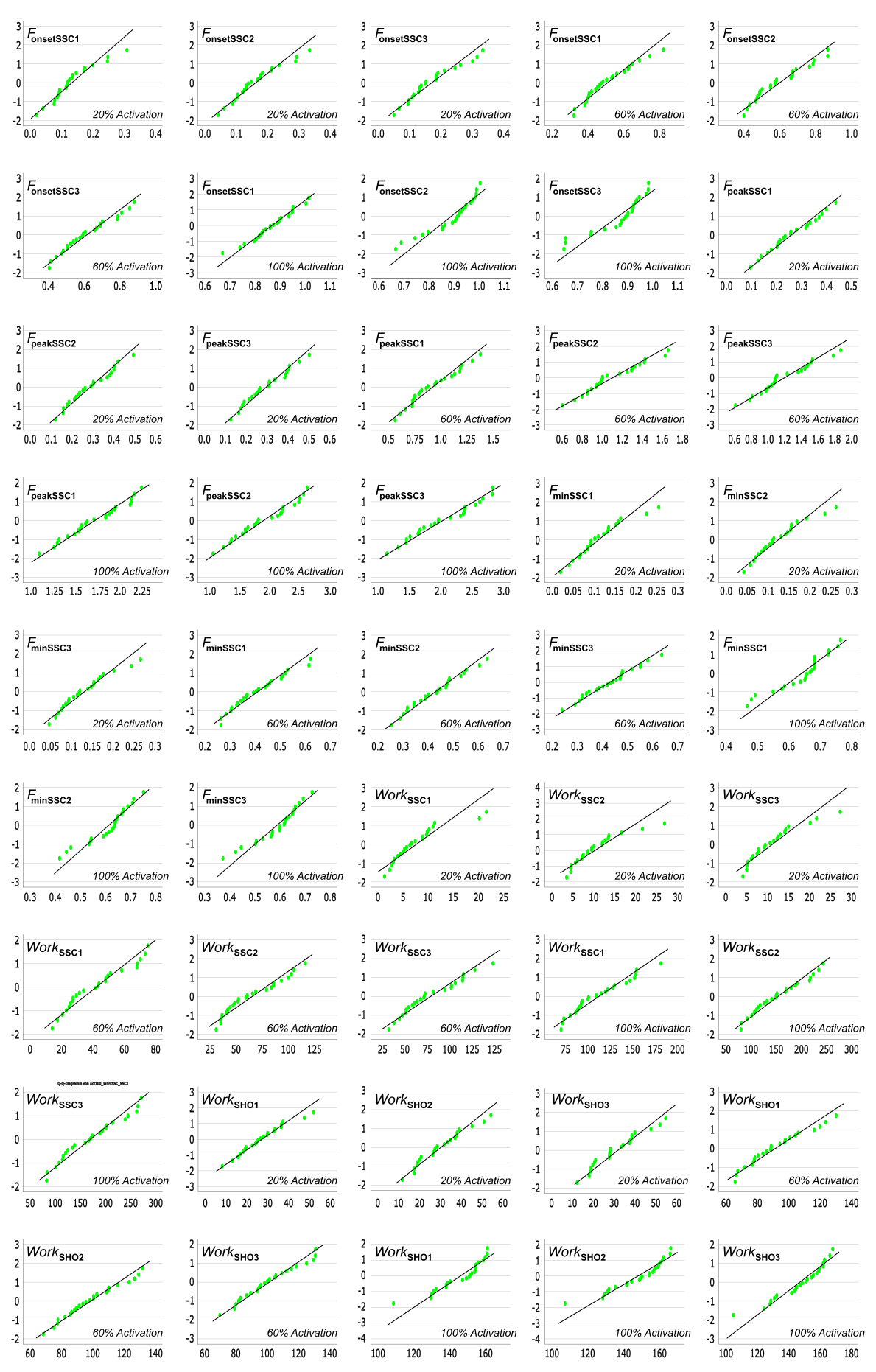


Figure S3. QQ plots for every parameter tested. Note the differently scaled x-axis and y-axis in every plot. The x-axis represents the sample quantiles while the y-axis represents the theoretical quantiles. QQ Plots hint that normality is questionable for 8 of 45 cases.
